# Proactive therapeutic drug monitoring of biologic drugs in patients with inflammatory bowel disease, inflammatory arthritis, and psoriasis: systematic review and meta-analysis

**DOI:** 10.1136/bmjmed-2024-000998

**Published:** 2024-10-28

**Authors:** Dena Zeraatkar, Tyler Stacy Pitre, Sarah Kirsh, Tanvir Jassal, Michael Ling, Muizz Hussain, Rachel J Couban, Leticia Kawano-Dourado, Eirik K Kristianslund, Per Olav Vandvik

**Affiliations:** 1 Department of Anesthesia, McMaster University, Hamilton, ON, Canada; 2 Department of Health Research Methods, Evidence, and Impact, McMaster University, Hamilton, ON, Canada; 3 Division of Respirology, Department of Medicine, University of Toronto, Toronto, ON, Canada; 4 MAGIC Evidence Ecosystem Foundation, Oslo, Norway; 5 Hcor Research Institute, Hcor Hospital, Sao Paulo, Brazil; 6 Pulmonary Division, Heart Institute (InCor), University of Sao Paulo, Sao Paulo, Brazil; 7 Center for treatment of Rheumatic and Musculoskeletal Diseases (REMEDY), Diakonhjemmet Hospital, Oslo, Norway; 8 Institute of Health and Society, University of Oslo Faculty of Medicine, Oslo, Oslo, Norway; 9 Department of Medicine, Lovisenberg Diakonale Hospital, Oslo, Oslo, Norway

**Keywords:** Rheumatology, Inflammatory bowel diseases, Psoriasis

## Abstract

**Objective:**

To address the efficacy and safety of proactive therapeutic drug monitoring of biologic drugs for patients with inflammatory bowel disease, inflammatory arthritis, and psoriasis.

**Design:**

Systematic review and meta-analysis.

**Data sources:**

Medline, Embase, Central, and CINAHL, from database inception to 23 May 2024.

**Eligibility criteria for selecting studies:**

Trials including people with inflammatory bowel disease, inflammatory arthritis, and psoriasis were selected. Selected trials also randomly assigned people to either proactive therapeutic drug monitoring of tumour necrosis factor-alpha inhibitors or other biologic drugs in the intervention group, and to either no therapeutic drug monitoring or standard care in the control group. Reviewers worked independently and in duplicate to screen search records and collect data from eligible trials. For each outcome, a frequentist, pairwise, random effects meta-analysis was done and the certainty of evidence was assessed using GRADE (grading of recommendations, assessment, development, and evaluations).

**Results:**

Of 10 eligible trials identified, reporting on 2383 patients, two investigated induction with infliximab (533 patients), four assessed maintenance with infliximab (901 patients), and three assessed maintenance with adalimumab (710 patients). One trial was of maintenance with infliximab, adalimumab, and etanercept (239 patients). For patients who had induction with infliximab, the effects of proactive therapeutic drug monitoring on remission and adverse events were uncertain. Low certainty evidence suggested that proactive therapeutic drug monitoring may have little or no effect on disease activity, physical function, mental health, and quality of life. For patients who had maintenance with infliximab, low certainty evidence suggested that proactive therapeutic drug monitoring may increase the proportion of patients who had sustained disease control or remission (relative risk 1.26 (95% confidence interval (CI) 1.14 to 1.40), absolute risk difference of 146 more per 1000 patients treated for one year (95% CI 78 to 224). Additionally, this treatment and monitoring may reduce disease worsening, and may have little or no effect on disease activity, physical function, mental health, and quality of life. The effects of proactive therapeutic drug monitoring of infliximab on adverse events and formation of anti-drug antibodies were uncertain. For patients who had maintenance with adalimumab, the effects of proactive therapeutic drug monitoring were uncertain.

**Conclusion:**

Proactive therapeutic drug monitoring of infliximab during maintenance may help patients to have sustained disease control or remission. No compelling evidence supported the effectiveness of proactive therapeutic drug monitoring of infliximab during induction or proactive therapeutic drug monitoring of adalimumab during maintenance.

**Systematic review registration:**

https://osf.io/x4m28/.

**WHAT IS ALREADY KNOWN ON THIS TOPIC**

- Biologic drugs, such as infliximab and adalimumab, have improved the care of patients with immune mediated inflammatory diseases
- Observational evidence shows that higher biologic drug concentrations during treatment are associated with better outcomes
- Proactive therapeutic drug monitoring—measuring drug concentrations and anti-drug antibodies at timed intervals to optimise individual patient dosage regimens and achieve target drug serum levels—may optimise serum drug concentrations and thus patient outcomes and prevent undertreatment and anti-drug antibody formation

**WHAT THIS STUDY ADDS**

- For patients undergoing maintenance with infliximab, low certainty evidence suggests that proactive therapeutic drug monitoring may increase the proportion of patients who had sustained disease control or remission
- Compelling evidence does not support the effectiveness of proactive therapeutic drug monitoring of infliximab or adalimumab during induction or adalimumab during maintenance

**HOW THIS STUDY MIGHT AFFECT RESEARCH, PRACTICE, OR POLICY**

- In clinical practice, proactive therapeutic drug monitoring of infliximab during maintenance treatment could be incorporated as standard practice to increase the likelihood of sustained disease control or remission
- Future research should focus on therapeutic drug monitoring to improve patient outcomes, particularly in the maintenance phases of treatment

## Introduction

 Inflammatory bowel disease (eg, ulcerative colitis and Crohn's disease), inflammatory arthritis (eg, rheumatoid arthritis, spondylarthritis, and psoriatic arthritis), and psoriasis are chronic immune mediated inflammatory diseases with related pathophysiology and systemic inflammation and cause a high burden on patients and healthcare systems.^1 2^ Patients experience pain, have progressive disability, are admitted to hospital, and need surgical intervention. These diseases are typically treated with glucocorticoids, broad spectrum immunomodulators, or biologic drugs, or a combination.^3^,^7^

Biologic drugs, such as tumour necrosis factor inhibitors (eg, infliximab and adalimumab), and other similar biologics that target cytokines, B cells, and T cells have improved the care of patients with immune mediated inflammatory diseases.^8^,^10^ These drugs reduce inflammation and structural damage to organs, consequently alleviating symptoms, disability, comorbidities, and mortality.^11 12^ Despite these advances, some patients do not respond to treatment, and about 50% of initial responders eventually had inadequate disease control, some of which could be explained by accelerated drug clearance or the formation of neutralising anti-drug antibodies.^13^

Observational evidence shows that higher biologic drug concentrations during treatment are associated with better outcomes.^14^,^20^ However, even among patients on the same dose of biologic drugs, serum drug concentrations vary largely.^21^ Thus, proactive therapeutic drug monitoring (ie, measuring drug concentrations and anti-drug antibodies at timed intervals to optimise individual patient dosage regimens and meet target drug serum levels) might improve outcomes and prevent undertreatment and anti-drug antibody formation (box 1).^20 22^ This approach could be more effective compared with reactive therapeutic drug monitoring, where drug concentrations are only measured in response to disease worsening, non-response, or the occurrence of an adverse event.^20 22^

Box 1 **Key term**

**Proactive therapeutic drug monitoring**

Scheduled measurement of serum drug concentrations and anti-drug antibodies to optimise individual patient dosage regimens and achieve target drug serum levels irrespective of symptoms or disease worsening.^20 22^

**Reactive therapeutic drug monitoring**

Measurement of drug concentrations and anti-drug antibodies in response to disease worsening, non-response, or occurrence of an adverse event to optimise patient outcomes.^20 22^ Reactive therapeutic drug monitoring is considered standard care.

**Induction of treatment**

Initiation and use of biologic drugs when patients are in a state of active disease, with the aim of treatment to achieve disease control, preferably by reaching a state of remission. The duration of induction treatment can vary across diseases. Induction differs from maintenance on the basis of treatment objective: induction aims to achieve remission or disease control, while maintenance focuses on sustaining that state.

**Maintenance of treatment**

Use of biologic drugs when patients are in a state of disease control to avoid disease worsening.

Results from randomised trials investigating therapeutic drug monitoring, however, have been mixed,^23 24^ leading to differing guideline recommendations^3 5 7 21 25^ and inconsistent clinical practice.^26^,^29^ Whether proactive therapeutic drug monitoring is effective during treatment induction is also unclear when biologic drugs are first initiated while patients are in a state of active disease, or during maintenance, when patients have disease control, or during both phases of treatment (box 1).

We present a systematic review and meta-analysis of randomised trials addressing the comparative efficacy and safety of proactive therapeutic drug monitoring of biologics during induction or maintenance in patients with immune mediated inflammatory diseases, including inflammatory bowel disease, inflammatory arthritis, and psoriasis. This systematic review is part of the BMJ Rapid Recommendations project, which is a collaborative effort from the MAGIC Evidence Ecosystem Foundation (www.magicevidence.org) and *The BMJ* to produce trustworthy recommendations in response to emerging practice changing evidence (box 2). This BMJ Rapid Recommendation is in response to new evidence from two randomised trials investigating therapeutic drug monitoring for inflammatory bowel disease.^23 24^ The parallel guideline panel, comprised of patient partners, rheumatologists, gastroenterologists, a dermatologist, a biochemist, a pharmacologist, and methodologists, who helped to define the research question and the scope of the review.

Box 2 **Linked articles in this BMJ Rapid Recommendations cluster**

**Practice article**: Kawano-Dourado L, Kristianslund EK, Zeraatkar D, et al. Proactive therapeutic drug monitoring of biologic drugs in adult patients with inflammatory bowel disease, inflammatory arthritis, or psoriasis: a clinical practice guideline. *BMJ* 2024;387:e079830

A clinical practice guideline from the rapid recommendations process

**Research article**: Zeraatkar D, Pitre T, Kirsh S, et al. Proactive therapeutic drug monitoring of biologic drugs inpatients with inflammatory bowel disease, inflammatoryarthritis, and psoriasis: systematic review and meta-analysis. *BMJMED* 2024;3:e000998

Systematic review and meta-analysis of all available randomised trials that assessed the efficacy and safety of proactive therapeutic drug monitoring of biologic drugs for patients with inflammatory bowel disease, inflammatory arthritis, and psoriasis

**MAGICapp version** (https://app.magicapp.org/#/guideline/8158)

Expanded version of the results with multilayered recommendations, evidence summaries, and decision aids for use across electronic devices

## Methods

We registered our protocol on Open Science Framework in June 2023 (https://osf.io/x4m28/). We report our study according to the PRISMA (preferred reporting items for systematic reviews and meta-analyses) checklist.^30^

### Eligibility criteria

Eligible studies randomised adults, children, or adolescents with inflammatory bowel disease (ulcerative colitis, Crohn's disease), inflammatory arthritis (rheumatoid arthritis, spondylarthritis, psoriatic arthritis), and psoriasis—diseases with related pathophysiology marked by systemic inflammation—to either proactive therapeutic drug monitoring of any type of biologic drugs (referred to here as therapeutic drug monitoring) or no therapeutic drug monitoring or reactive therapeutic drug monitoring (referred to here as standard care), during either induction or maintenance of treatment.

We excluded non-randomised studies, systematic and scoping reviews, trials of therapeutic drug monitoring of antibiotics and other types of drugs, and trials of therapeutic drug monitoring in patients with immune mediated inflammatory diseases without rheumatological involvement (eg, asthma, cancer, neurodegenerative disease). We did not restrict eligibility based on publication status (ie, preprint or publication) or by date or language of publication.

### Search strategy

An experienced medical librarian devised a search strategy for MEDLINE, EMBASE, CENTRAL, and CINAHL for randomised trials from inception to May 2024 (online supplement 1), without any restrictions on language or publication status. Our search combined terms related to inflammatory bowel disease, inflammatory arthritis, and psoriasis, biologics, and a filter for randomised trials. We supplemented our search by reviewing the reference lists of relevant reviews and soliciting the parallel BMJ Rapid Recommendations guideline panel for trials that might have been missed from our search.^2531^,^33^

### Screening

Following training and calibration exercises to ensure sufficient agreement, pairs of reviewers, working independently and in duplicate, screened the titles and abstracts of search records and subsequently the full texts of records deemed eligible at the title and abstract screening stage using Covidence (https://www.covidence.org), an online systematic review software. Reviewers resolved discrepancies by discussion or, when necessary, by adjudication with a third reviewer.

### Data extraction

Following training and calibration exercises to ensure sufficient agreement, pairs of reviewers, working independently and in duplicate, extracted data from eligible trials using a pilot tested, structured data extraction form in Excel (Microsoft Office 2019). We collected data on trial characteristics (eg, funding, country), patient characteristics (eg, age, sex, types of immune mediated inflammatory diseases, years since diagnosis, immunosuppressive treatment), intervention characteristics (eg, drug, target dose, algorithm for dose adjustment), and outcomes of interest.

In selecting our outcomes, we prioritised patient importance. Our choice of outcomes was informed by published core outcome sets for immune mediated inflammatory diseases and by input from the parallel BMJ Rapid Recommendations guideline panel.^34 35^ Patient important outcomes include sustained remission, sustained disease control, remission, change in disease activity, quality of life, physical function, mental health, work disability, serious adverse events, adverse events leading to discontinuation, and formation of anti-drug antibodies.

We used established definitions of outcomes. We defined remission as reduced disease activity under a set limit according to disease specific activity scores, sustained remission as remission during the study period or some prespecified period, and sustained disease control as a state without disease worsening defined by disease specific activity scores or as disease worsening leading to a major change in treatment (switching to another biologic drug, adding an immunosuppressive drug including a systemic glucocorticoid, or increasing the infliximab dose for clinical reasons).^23 24^ We considered the following disease specific activity scores: the Disease Activity Score in 28 Joints (DAS28) score for rheumatoid arthritis and psoriatic arthritis (<2.6 for remission; increase in score≥1.2 and a minimum score of 3.2 for worsening),^36 37^ the Bath Ankylosing Spondylitis Disease Activity Index (BASDAI) for spondylarthritis (<1.3 for remission; increase in score≥1.1 and a minimum score of 2.1 for worsening),^38 39^ Partial or Full Mayo Score for ulcerative colitis (≤2 with no subscore>1 for remission; increase in score≥3 and minimum score of 5 for worsening),^40^ Harvey-Bradshaw Index (HBI) or the Crohn's Disease Activity Index (CDAI) for Crohn's disease (HBI≤4 for remission; increase in HBI score≥4 and a minimum score of 7 for worsening; CDAI<150 for remission),^41^,^45^ and the Psoriasis Area and Severity Index (PASI) for psoriasis (≤4 for remission; increase in score≥3 and minimum score of 5 for worsening).^46^ These scoring systems and thresholds are widely recognised and represent systems and thresholds used to measure disease activity both in clinical practice and in research.^2324 37 47^,^50^

Both sustained remission and sustained disease control indicate disease activity. As typically defined, all patients who remain in sustained remission also achieve sustained disease control. Patients in sustained remission are in remission during the whole period while patients in sustained disease control might experience disease activity above the remission threshold, as long as their disease activity does not significantly worsen from baseline.

For dichotomous outcomes, we extracted the number of patients and events in each arm, and, for continuous outcomes, for each arm, we extracted the number of patients, a measure of central tendency, and a measure of variability. We preferentially extracted results from intention-to-treat analyses with complete case data at the longest reported time of follow-up at which patients were still undergoing proactive therapeutic drug monitoring.

Based on input from the parallel BMJ Rapid Recommendations guideline panel, we hypothesised that therapeutic drug monitoring is more beneficial in rheumatic arthritis and inflammatory bowel diseases owing to their immunogenic nature and higher likelihood of formation of anti-drug antibodies, in patients without concurrent immunosuppression due to the lower likelihood of formation of anti-drug antibodies, and in patients with a history of anti-drug antibodies. Hence, when reported, we extracted outcome data stratified by type of disease, concomitant immunosuppression, and number of biologics that patients had previously used.

### Risk of bias

Following training and calibration to ensure sufficient agreement, reviewers worked independently and in duplicate, to assess risk of bias using a modified Cochrane RoB (risk of bias) 2.0 tool.^51 52^ The Cochrane RoB 2.0 tool rates risk of bias of results as either "high," "some concerns," and "low" across the following domains: bias arising from the randomisation process, bias due to departures from the intended intervention, bias due to missing outcome data, bias in measurement of the outcome, and bias in selection of the reported results. To assess the risk of bias due to deviations from the intended intervention, we considered the effect of assignment rather than adherence to the intervention—because this effect is likely to be the observed effect in clinical settings and of the greatest interest to evidence users.

The modified version of the Cochrane RoB 2.0 tool rates risk of bias as either "low risk of bias," "some concerns (probably low risk of bias)," "some concerns (probably high risk of bias)," and "high risk of bias" (online supplement 2). The first two categories are combined as "low risk of bias" and the last two categories are combined as "high risk of bias." This approach simplifies decisions about whether most of the evidence comes from studies at high or low risk of bias and when to rate down the certainty of evidence for risk of bias. The modified version of the Cochrane RoB 2.0 tool includes the same domains as the original tool but the guidance is tailored to the question being studied (eg, removing guidance for assessing risk of bias of adhering to the intervention).

We presumed that failure to blind patients and healthcare providers might lead to differences in care across trial arms, such as patients' likelihood to report disease symptoms or physicians' propensity to monitor patients and modify drug dosages.^53^ Such variations in care might confound the effects of therapeutic drug monitoring. For this reason, we considered trials without blinding of patients or healthcare providers to be at high risk of bias for deviations from the intended intervention. Reviewers resolved discrepancies by discussion and if necessary, by adjudication with a third reviewer.

### Data synthesis and analysis

Based on input from the parallel BMJ Rapid Recommendations guideline panel, we anticipated that the effects of therapeutic drug monitoring will be different based on its use during induction and maintenance and the biologic drug for which it is used. For example, during induction, drugs are given at higher doses and during a period of disease activity, which might influence the effectiveness of therapeutic drug monitoring. Similarly, infliximab and adalimumab could have different degrees of immunogenicity, which might also influence the effectiveness of therapeutic drug monitoring.^4^ There might also be increased immunogenicity with intravenous, chimeric drugs such as infliximab compared with fully human proteins such as adalimumab that are given subcutaneously.^4^ Hence, we stratified analyses based on phase of treatment and biologic drug.

Conversely, following extensive deliberation, the parallel guideline panel decided that inflammatory bowel disease, inflammatory arthritis, and psoriasis can be grouped together in analyses. This decision aligns with GRADE guidance that advises subgroups to be combined unless there is compelling evidence to suspect differences.^35^ The panel considered that current research and the best available evidence do not indicate heterogeneity in the effects of therapeutic drug monitoring among these diseases, and evidence suggests that these diseases share similar pathophysiology.^23 24^

For each comparison and outcome, we performed frequentist, random effects meta-analysis using the restricted maximum likelihood estimator to pool the results of individual trials together. We reported relative risks and associated 95% confidence intervals (CI) for dichotomous outcomes and mean differences and associated 95% CIs for continuous outcomes.

To facilitate interpretation, for all dichotomous outcomes, we calculated the risk differences per 1000 patients by multiplying the relative risk obtained from meta-analyses with an estimate of the risk of the outcome with standard care. We estimated the risk of the outcome with standard care by calculating a median risk across control groups of all relevant trials. Since the probability of outcomes probably varies on the basis of the specific disease, when available, we preferentially used data from trials that included representation across all eligible immune mediated conditions.^54^ We used the same baseline risk estimates for infliximab and adalimumab based on evidence of suggesting that they have similar efficacy and safety.^55^,^59^ The parallel guideline panel provided input on the applicability of the standard care group risks.

We summarised heterogeneity using the I^2^ statistic and interpreted an I^2^ value of 0-40% as not important, 30-60% as moderate heterogeneity, 50-90% as substantial heterogeneity, and 75-100% as considerable heterogeneity.^60^ The I^2^ value is prone to misinterpretation because even small degrees of unimportant inconsistency can translate to high I^2^ values if estimates from studies are highly precise.^61 62^ Hence, we also considered the absolute magnitude of differences in effect estimates across studies. For analyses with 10 or more trials, we planned to test for publication bias by visually inspecting funnel plots and performing Egger's tests.^63^ None of our analyses, however, included 10 or more trials.

To test for subgroup effects based on risk of bias, type of disease, concomitant immunosuppression, and use of previous biologic drugs, we did subgroup analyses and evaluated the credibility of subgroup effects using the ICEMAN tool.^64^ We performed all analyses using the meta and metafor packages in R (version 4.1.2).^65 66^

### Assessment of the certainty (quality) of evidence and reporting of results

We assessed the certainty of evidence using the GRADE approach.^67^ For each comparison and outcome, we rated the certainty as either high, moderate, low, or very low based on considerations of risk of bias (limitations in study designs that could lead to systematic understimation or overestimation of treatment effects), inconsistency (differences in results across studies asking the same or similar questions), indirectness (differences between the question of interest and the questions asked in studies), imprecision (random error), and publication bias (the propensity for studies with interesting results to be published over those with uninteresting results, or to be published faster or in journals with higher visibility). High certainty evidence suggests that the estimated effect from the systematic review and meta-analysis is likely close to the true effect, and low or very low certainty evidence suggests that the estimated effect might be substantially different from the true effect.

To make judgments about imprecision, we used a minimally contextualised approach, which considers only whether CIs include a minimally important effect and does not consider the magnitude of plausible effects.^68^ Judgments about minimally important effects were made on the basis of published minimally important differences or by consensus of the authors and the parallel guideline development group. The final assessment of certainty was fully contextualised by the parallel BMJ Rapid Recommendations guideline panel to formulate recommendations. We reported results using GRADE simple language summaries (ie, describing high certainty evidence with declarative statements, moderate certainty evidence with "probably," low certainty evidence with "may" and very low indicated by "very uncertain").^69^

### Deviations from protocol

We initially intended to pool trials investigating therapeutic drug monitoring of all biologic drugs. Based on input from the parallel guideline panel regarding the distinct immunogenicity of these drugs and the potential for varying effects of therapeutic drug monitoring depending on the drug type, we instead stratified analyses by biologic drug.

### Patient and public involvement

The parallel BMJ Rapid Recommendations guideline panel included two patient partners, who provided input on the scope of the systematic review, including the outcomes of interest.

## Results

**Figure 1:**
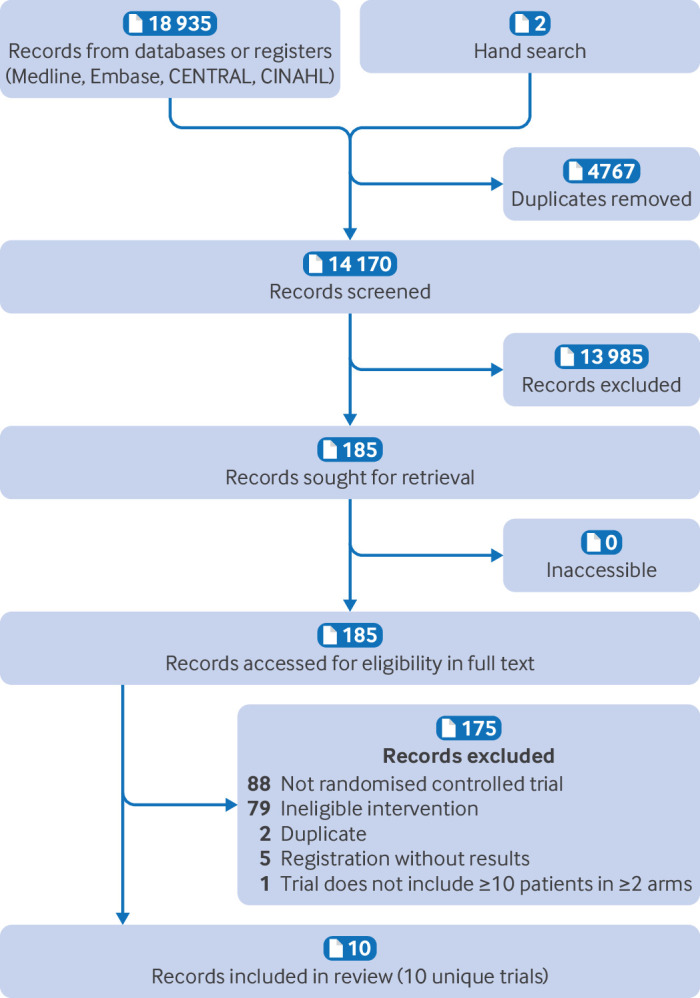
Study selection

Our search yielded 18 937 references, of which 10, including 2383 patients, proved eligible.^923 24 70^,^76^ Figure 1 presents the study selection process.

### Trial and patient characteristics

Table 1 presents trial and patient characteristics and online supplement 3 presents additional characteristics. All trials were published in English, used parallel group designs, and were published in peer reviewed journals. Results from one trial were only available as a conference abstract.^75^ All but two trials were conducted in western Europe; the exceptions were conducted in Israel and South Korea.^74 75^ Most trials reported on the effects of proactive therapeutic drug monitoring in middle aged adults, with two trials including children and adolescents.^74^ ^75^ Two trials addressed induction with infliximab (533 patients),^23 70^ four trials maintenance with infliximab (901 patients),^9 24 71 75^ and three trials maintenance with adalimumab (710 patients).^72^,^74^ One trial addressed maintenance with infliximab, adalimumab, and etanercept (239 patients). For those patients with specific inflammatory disease reported, the review included 258 (11.1%) with rheumatoid arthritis, 347 (14.9%) with spondylarthritis, 92 (4.0%) with psoriatic arthritis, 708 (30.4%) with ulcerative colitis, 863 (37.1%) with Crohn's disease, and 59 (2.5%) with psoriasis.

**Table 1:** Trial and patient characteristics

**Study (No of patients)**	**Registration**	**Country**	**Male (%)**	**Age (median; years) and trial group**	**Drug, induction, or maintenance**	**Conditions (%**)	**Duration of disease (years) by trial group**
TAILORIX^70^ (n=122)	2011-003038-14	Belgium, France, Netherlands	41.8	29.1 (proactive TDM (2.5 mg dose escalation)), 30.2 (proactive TDM (5 mg dose escalation)),28.7 (SC)	Infliximab or induction	Crohn's disease (100)	0.4 (TDM, 2.5 mg), 1 (TDM, 5 mg), 0.5 (SC)
NOR-DRUM-A^23^ (n=411)	NCT03074656	Norway	48.99	44 (TDM), 44 (SC)	Infliximab or induction	Rheumatoid arthritis (20.1), spondylarthritis (29.4), psoriatic arthritis (10.55), ulcerative colitis (20.1), Crohn's disease (14.32), psoriasis (5.53)	3.5 (TDM), 3.8 (SC)
Pailot^74^ (n=78)	NCT02256462	Israel	70.51	14.31*	Adalimumab or maintenance	Crohn's disease (100)	0.58 (proactive TDM), 0.41 (reactive TDM)
Casteele (TAXIT), 2015 (n=251)	2011-002061-38	Belgium	54.98	41 (TDM), 42 (SC)	Infliximab or maintenance	Ulcerative colitis (27.49), Crohn's disease (54.98)	12 (TDM), 12.5 (SC)
SERENE CD^72^ (n=184)	NCT02065570	Austria,Belgium,Canada, Czech Republic,Denmark,France,Germany, Hungary, Italy, Netherlands, Poland, Romania,Slovakia,Spain,Switzerland, Ukraine, UK, US	52.17	34 (proactive TDM), 32 (SC)	Adalimumab or maintenance	Crohn's disease (100)	6.3
Kang 2024 (n=112)	NR	South Korea	NR	Children	Infliximab or maintenance	Crohn's disease (100)	NR
SERENE UC^73^ (n=448)	NCT02065622	Austria, Belgium, Canada, Czech Republic, Denmark,France,Germany, Hungary,Israel,Italy, Netherlands, Poland, Romania, Slovakia, Spain, Switzerland, Ukraine, UK, US	54.99	37 (TDM), 40 (SC every week), 37(SC every other week),	Adalimumab or maintenance	Ulcerative colitis (100)	6.2 (TDM), 6.7 (SC every week), 7.8 (SC every other week)
Pfeffer-Jensen, 2022 (n=239)	EUDRA-CT2015-004173-32	Denmark	42.26	51.15*	Infliximab, adalimumab, etanercept or maintenance	Rheumatoid arthritis (41.42), spondylarthritis (38.49), psoriatic arthritis (20.08)	10.91
Strik, 2020 (PRECISION) (n=80)	NCT02453776	Netherlands	33.75	38 (TDM), 37 (SC)	Infliximab or maintenance	Ulcerative colitis (17.5), Crohn's disease (82.5)	10 (TDM), 10.6 (SC)
NOR-DRUM-B^23^ (n=458)	NCT03074656	Norway	52.42	44.85*	Infliximab or maintenance	Rheumatoid arthritis (17.4), spondylarthritis (30.4), psoriatic arthritis (11.67), ulcerative colitis (17.84), Crohn's disease (14.54), psoriasis (8.15)	6.2 (TDM), 5.3 (SC)

* Mean age.

NR, Not reported; SC, Standard care; TDM, Therapeutic drug monitoring.

One trial first randomised patients with ulcerative colitis during induction to higher versus lower doses of adalimumab.^73^ This initial phase of the trial during induction of patients was not eligible because it did not test therapeutic drug monitoring. The trial, however, subsequently reassigned patients randomly to adalimumab 40 mg every week, adalimumab 40 mg every other week, or adalimumab with therapeutic drug monitoring. The trial reports efficacy outcomes restricted to patients who achieved remission during induction but reports safety outcomes for the total population regardless of remission status. We restricted descriptive characteristics of the study population to those who had reached remission during induction but reported safety outcomes for the total trial population.

In another trial of adults with active Crohn's disease, researchers randomised patients at 14 weeks after the first dose of infliximab to proactive therapeutic drug monitoring or standard care.^70^ We grouped this trial with those trials reporting on induction because it initially recruited patients with active disease and we deemed 14 weeks insufficient for most patients to achieve remission before randomisation.

At baseline, nearly half of patients were using immunosuppressive treatment and a quarter were using steroids. Trials reported about 10% of patients to have previously used other biologics. Four trials compared proactive therapeutic drug monitoring with dosing based on clinical judgment,^9 23 24 75^ one with reactive therapeutic drug monitoring,^74^ three with treatment without dose adaptations,^71 73^ and two with dose adaptations according to predefined clinical criteria.^70 72^

Online supplement 3 describes the implementation of therapeutic drug monitoring, including the frequency of therapeutic drug monitoring, target drug thresholds, adjustments to dose or interval at which drugs were given, the range of allowable doses and intervals, and thresholds of anti-drug antibody concentrations for discontinuing the drug.

### Risk of bias

**Figure 2:**
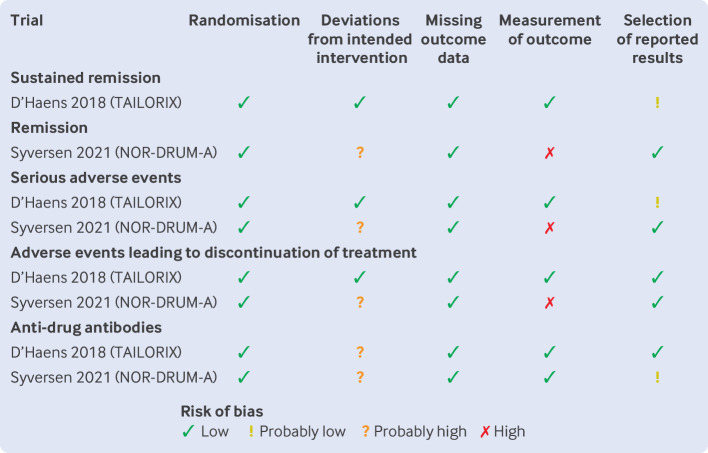
Risk-of-bias judgments for trials reporting on the effects of therapeutic drug monitoring during induction with infliximab

Figures2  3 present ratings of risk of bias for trials addressing induction and remission, respectively.

**Figure 3:**
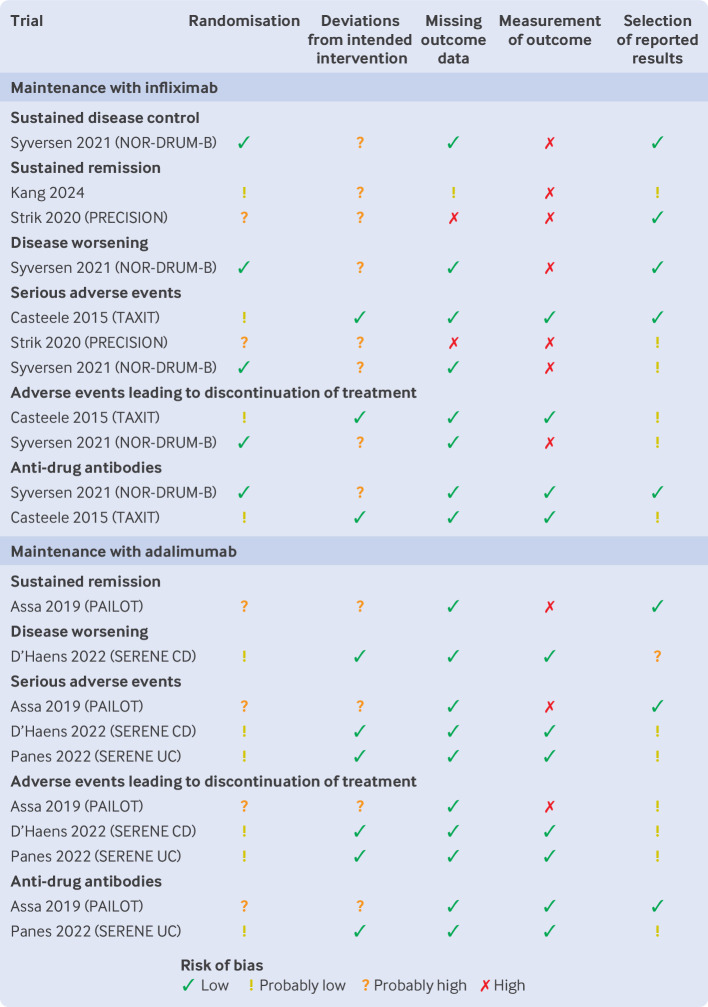
Risk-of-bias judgments for trials reporting on the effects of therapeutic drug monitoring during maintenance

Most evidence was rated at high risk of bias, owing to concerns about differences in care and co-interventions and differences in measurement of outcomes arising from the open label design of trials.

### Therapeutic drug monitoring for induction

Two trials, including 533 patients, reported on the effects of proactive therapeutic drug monitoring during induction with infliximab.^23 70^ One trial (122 patients) included only patients with Crohn's disease, and the second trial (411 patients) included patients with rheumatoid arthritis, spondylarthritis, psoriatic arthritis, ulcerative colitis, Crohn's disease, and psoriasis. We did not identify any trials that looked at the effects of proactive therapeutic drug monitoring during induction with other biologic drugs.

Table 2 presents the results of meta-analyses comparing proactive therapeutic drug monitoring of infliximab with standard care, and online supplement 4 presents forest plots. Low certainty evidence suggested that proactive therapeutic drug monitoring with infliximab during induction might have had little or no effect on sustained remission, disease activity, quality of life, physical function, and mental health.

**Table 2:** Summary of findings of therapeutic drug monitoring of infliximab during induction of treatment

Outcome and timeframe	Study results and measurements	Absolute effect estimates		Certainty (quality) of evidence	Summary
		Standard care	Proactive TDM		
Remission at 30 weeks	Relative risk 0.95 (95% CI 0.79 to 1.15), based on data from 398 participants in one study	530 per 1000	504 per 1000	Very low (owing to serious risk of bias, serious imprecision, serious indirectness*)	We are uncertain of the effects of proactive therapeutic drug monitoring of infliximab on achieving remission
		Difference: 26 fewer per 1000 (95% CI 111 fewer to 80 more)			
Sustained remission during 54 weeks	Relative risk 0.76 (95% CI 0.46 to 1.26), based on data from 122 participants in one study	400 per 1000	304 per 1000	Low (owing to serious imprecision, serious indirectness†)	Proactive therapeutic drug monitoring of infliximab may have little or no effect on achieving sustained remission
		Difference: 96 fewer per 1000 (95% CI 216 fewer to 104 more)			
Serious adverse events, 30-54 weeks	Relative risk 1.15 (95% CI 0.76 to 1.73), based on data from 520 participants in two studies	100 per 1000	115 per 1000	Very low (owing to serious risk of bias, serious imprecision, serious indirectness‡	We are uncertain of the effects of proactive therapeutic drug monitoring of infliximab on serious adverse events
		Difference: 15 more per 1000 (95% CI 24 fewer to 73 more)			
Adverse events leading to discontinuation of treatment, 30-54 weeks	Relative risk 0.68 (95% CI 0.33 to 1.39), based on data from 520 participants in two studies	80 per 1000	54 per 1000	Very low (owing to serious risk of bias, serious imprecision, serious indirectness‡)	We are uncertain of the effects of proactive therapeutic drug monitoring of infliximab on adverse events leading to discontinuation of treatment
		Difference: 26 fewer per 1000 (95% CI 54 fewer to 31 more)			
Anti-drug antibodies, 20-54 weeks	Relative risk 1.14 (95% CI 0.78 to 1.68), based on data from 520 participants in two studies	170 per 1000	194 per 1000	Very low (owing to serious risk of bias, serious imprecision, serious indirectness‡)	We are uncertain of the effects of proactive therapeutic drug monitoring of inxlimab on anti-drug antibodies
		Difference: 24 more per 1000 (95% CI 37 fewer to 116 more)			
Disease worsening	No eligible trials				
Change in disease activity, 30 weeks	Measured by patient's global assessment of disease activity scale 0-100§, based on data from 398 participants in one study	Mean difference: 3.7 higher (95% CI 0.6 lower to 8.0 higher)		Low (owing to serious risk of bias, serious indirectness*)	Proactive therapeutic drug monitoring of infliximab may have little or no effect on disease activity
Change in disease activity, 30 weeks	Measured by physician's global assessment of disease activity scale 0-100§, based on data from 398 participants in one study	Mean difference: 1.7 higher (95% CI 1.8 lower to 5.2 higher)		Low (owing to serious risk of bias, serious indirectness*)	Proactive therapeutic drug monitoring of infliximab may have little or no effect on disease activity
Quality of life, 30 weeks	Measured by EQ-5D scale 0-1¶, based on data from 398 participants in one study	Mean 0.5	Mean 0.5	Low (owing to serious risk of bias, serious indirectness*)	Proactive therapeutic drug monitoring of infliximab may have little or no effect on quality of life
		Mean difference: 0 lower (95% CI 0 lower to 0.1 higher)			
Physical function, 30 weeks	Measured by SF-36 physical component scale 0-100¶, based on data from 398 participants in one study	Mean 38.8	Mean 38.8	Low (owing to serious risk of bias, serious indirectness*)	Proactive therapeutic drug monitoring of infliximab may have little or no effect on physical function
		Mean difference: 0 lower (95% CI 1.5 lower to 1.5 higher)			
Mental health, 30 weeks	Measured by SF-36 mental component scale 0-100¶, based on data from 398 participants in one study	Mean 45.2	Mean 46.1	Low (owing to serious risk of bias, serious risk of bias, serious indirectness*)	Proactive therapeutic drug monitoring of infliximab may have little or no effect on mental health
		Mean difference: 0.9 higher (95% CI 0.8 lower to 2.7 higher)			
Work disability	No eligible trials				

* Data come from a trial of patients with immune mediated inflammatory diseases. It is uncertain whether the effects of TDM are consistent across diseases.

† Data come from a trial of patients with Crohn's disease. It is uncertain whether the effects of TDM are consistent across diseases.

‡ Data come from a trial of patients with immune mediated inflammatory diseases and a trial of patients with Crohn's disease. It is uncertain whether the effects of TDM are consistent across diseases.

§ Higher score represents worse perceived disease activity or overall health.

¶ Where the highest score (1 or 100) represents the best possible health state.

CI, confidence interval; TDM, Therapeutic drug monitoring.

We rated down the certainty of evidence for risk of bias when the outcome was informed primarily by trials rated at high risk of bias and for imprecision when CIs included clinically important benefit, harm, and no or trivial effect. We also rated down the certainty of evidence for concerns related to indirectness across all outcomes because, while subgroup analyses suggest that the effects of therapeutic drug monitoring are consistent across immune mediated inflammatory diseases, estimates for the effect of therapeutic drug monitoring across subgroups are imprecise and the effects of therapeutic drug monitoring might vary across immune mediated inflammatory diseases. We are very uncertain of the effects of proactive therapeutic drug monitoring with infliximab on remission, serious adverse events, adverse events leading to discontinuation of treatment, and the formation of anti-drug antibodies.

We anticipated that the effects of therapeutic drug monitoring might be different in trials at low versus high risk of bias, based on disease, and in adults versus children. We did not find evidence that the effects of therapeutic drug monitoring were different in trials at low versus high risk of bias or based on disease, although effects across disease subgroups were imprecise (online supplements 5 and 6). Trials only investigated the effect of therapeutic drug monitoring in adults and so we do not have evidence on whether the effect of therapeutic drug monitoring may vary by age.

### Therapeutic drug monitoring during maintenance

#### Infliximab

Five trials, including 982 patients, reported on the effects of therapeutic drug monitoring of infliximab during maintenance.^9 24 71^ Three trials reported on patients with inflammatory bowel disease.^17 71^ One trial reported on patients with inflammatory arthritis^76^ and one trial reported on patients with rheumatoid arthritis, spondylarthritis, psoriatic arthritis, ulcerative colitis, Crohn's disease, and psoriasis.^24^ One trial reported on children.^75^

Table 3 presents the results of meta-analyses comparing proactive therapeutic drug monitoring of infliximab during maintenance with standard care, and online supplement 7 presents forest plots. Low certainty evidence suggests that proactive therapeutic drug monitoring of infliximab may increase the proportion of patients who experience sustained disease control or remission, may reduce disease worsening, and may have little or no effect on disease activity, physical function, mental health, and quality of life.

**Table 3:** Summary of findings of therapeutic drug monitoring of infliximab during maintenance

Outcome and timeframe	Study results and measurements	Absolute effect estimates		Certainty (quality) of evidence	Summary
		Standard care	Proactive TDM		
Sustained disease control or sustained remission, 52 weeks	Relative risk 1.26 (95% CI 1.14 to 1.40), based on data from 872 participants in four studies	560 per 1000	706 per 1000	Low (owing to serious risk of bias, serious indirectness*)	Proactive therapeutic drug monitoring of infliximab may increase the proportion of patients who experience sustained disease control or sustained remission
		Difference: 146 more per 1000 (95% CI 78 more to 224 more)			
Sustained disease control, 52 weeks	Relative risk 1.31 (95% CI 1.14 to 1.51), based on data from 454 participants in one study	560 per 1000	734 per 1000	Low (owing to serious risk of bias, serious indirectness†)	Proactive therapeutic drug monitoring of infliximab may increase the proportion of patients who experience sustained disease control
		Difference: 174 more per 1000 (95% CI 78 more to 286 more)			
Sustained remission, 52 weeks	Relative risk 1.20 (95% CI 1.03 to 1.40), based on data from 418 participants in three studies	625 per 1000	750 per 1000	Very low (owing to serious risk of bias, serious imprecision, serious indirectness‡)	We are uncertain of the effects of proactive therapeutic drug monitoring of infliximab on sustained remission
		Difference: 125 more per 1000 (95% CI 19 more to 250 more)			
Remission, 52 weeks	Relative risk 1.03 (95% CI 0.93 to 1.14), based on data from 688 participants in two studies	650 per 1000	670 per 1000	Very low (owing to serious risk of bias, serious imprecision, Due to serious indirectness§)	We are uncertain of the effects of proactive therapeutic drug monitoring of infliximab on remission
		Difference: 20 more per 1000 (95% CI 45 fewer to 91 more)			
Disease worsening, 52 weeks	Relative risk 0.6 (95% CI 0.46 to 0.78), based on data from 454 participants in one study	441 per 1000	265 per 1000	Low (owing to serious risk of bias, serious indirectness¶)	Proactive therapeutic drug monitoring of infliximab may reduce the proportion of patients who experience disease worsening
		Difference: 176 fewer per 1000 (95% CI 238 fewer to 97 fewer)			
Serious adverse events, 52 weeks	Relative risk 0.97 (95% CI 0.52 to 1.82), based on data from 785 participants in three studies	80 per 1000	78 per 1000	Very low (owing to serious risk of bias, very serious imprecision**)	We are uncertain of the effects of proactive therapeutic drug monitoring of infliximab on adverse events leading to discontinuation of medical treatment
		Difference: 2 fewer per 1000 (95% CI 38 fewer to 66 more)			
Adverse events leading to discontinuation of medical treatment, 52 weeks	Relative risk 0.96 (95% CI 0.06 to 15.19), based on data from 705 participants in two studies	50 per 1000	48 per 1000	Very low (owing to very serious imprecision, serious indirectness§)	We are uncertain of the effects of proactive therapeutic drug monitoring of infliximab on adverse events leading to discontinuation of treatment
		Difference: 2 fewer per 1000 (95% CI 47 fewer to 710 more)			
Anti-drug antibodies, 52 weeks	Relative risk 0.59 (95% CI 0.35 to 0.99), based on data from 659 participants in two studies	150 per 1000	89 per 1000	Very low (owing to serious risk of bias, serious imprecision, serious indirectness§)	We are uncertain of the effects of proactive therapeutic drug monitoring of infliximab on anti-drug antibodies
		Difference: 61 fewer per 1000 (95% CI 97 fewer to one fewer)			
Change in disease activity, 52 weeks	Measured by patient's global assessment scale 0-100††, based on data from 454 participants in one study	Mean difference: 0.9 higher (95% CI 2.4 lower to 4.2 higher)		Low (owing to serious risk of bias, serious indirectness¶)	Proactive therapeutic drug monitoring of infliximab may have little or no effect on disease activity
Change in disease activity, 52 weeks	Measured by physician's global assessment scale 0-100††, based on data from 454 participants in one study	Mean difference: 0.05 higher (95% CI two lower to three higher)		Low (owing to serious risk of bias, serious indirectness¶)	Proactive therapeutic drug monitoring of infliximab may have little or no effect on disease activity
Quality of life, 44 weeks	Measured by EQ-5D scale 0-1‡‡, based on data from 184 participants in one study	Mean 0.78	Mean 0.79	Low (owing to serious risk of bias, serious indirectness**)	Proactive therapeutic drug monitoring of infliximab may have little or no effect on quality of life
		Mean difference: 0.01 higher (95% CI 0.05 lower to 0.07 higher)			
Physical function, 52 weeks	Measured by SF-36 physical component scale 0-100‡‡, based on data from 454 participants in one study	Mean 48.2	Mean 48.7	Low (owing to serious risk of bias, serious indirectness¶)	Proactive therapeutic drug monitoring of infliximab may have little or no effect on physical function
		Mean difference: 0.5 higher (95% CI 1.6 lower to 0.6 higher)			
Mental health, 52 weeks	Measured by SF-36 mental component scale 0-100‡‡, based on data from 454 participants in one study	Mean 48.7	Mean 48.5	Low (owing to serious risk of bias, serious indirectness¶)	Proactive therapeutic drug monitoring of infliximab may have little or no effect on mental health
		Mean difference: 0.2 lower (95% CI 1.16 lower to 1.1 higher)			
Work disability	No eligible trials				

* Data come from two trials of patients with inflammatory bowel disease and a trial of patients with immune mediated inflammatory diseases. It is uncertain whether the effects of TDM are consistent across diseases.

† Data come from a trial of patients with immune mediated inflammatory diseases. It is uncertain whether the effects of TDM are consistent across diseases.

‡ Data come from two trials of patients with inflammatory bowel disease. It is uncertain whether the effects of TDM are consistent across diseases.

§ Data come from a trial of patients with inflammatory bowel disease and a trial of patients with immune mediated inflammatory diseases. It is uncertain whether the effects of TDM are consistent across diseases.

¶ Data come from a trial of patients with immune mediated inflammatory diseases. It is uncertain whether the effects of TDM are consistent across diseases.

** Data come from a trial of patients with Crohn's disease. It is uncertain whether the effects of TDM are consistent across diseases.

†† Higher score represents worse perceived disease activity or overall health.

‡‡ Where the highest score (1 or 100) represents the best possible health state.

CI, Confidence interval; TDM, Therapeutic drug monitoring.

We downgraded the certainty of evidence owing to serious concerns with risk of bias and indirectness. We rated down for indirectness because we were uncertain whether the effects of therapeutic drug monitoring are consistent across immune mediated inflammatory diseases. We are very uncertain of the effects of therapeutic drug monitoring of infliximab on remission, sustained remission, serious adverse events, adverse events leading to discontinuation of treatment, and anti-drug antibodies owing to serious concerns with risk of bias, imprecision, and indirectness. We did not find evidence addressing disease worsening or work or school absenteeism and disability.

We anticipated that the effects of therapeutic drug monitoring of infliximab might be different in trials at low versus high risk of bias and based on disease and age. We did not find evidence that the effects of therapeutic drug monitoring of infliximab were different in trials at low versus high risk of bias, based on disease, although effects across disease subgroups were imprecise, or based on age (online supplements 5,6, and 8).

#### Adalimumab

Four trials, including 789 patients, reported on the effects of therapeutic drug monitoring of adalimumab during maintenance.^72^,^74^ All three trials reported on patients with inflammatory bowel disease. One trial, with 78 patients, reported on children and adolescents.^74^ Table 4 presents results of meta-analyses comparing proactive therapeutic drug monitoring of adalimumab during maintenance with standard care, and online supplement 9 presents forest plots.

**Table 4:** Summary of findings of therapeutic drug monitoring of adalimumab during maintenance

Outcome and timeframe	Study results and measurements	Absolute effect estimates		Certainty (quality) of evidence	Summary
		Standard care	Proactive TDM		
Sustained disease control or sustained remission	Relative risk 1.72 (95% CI 1.2 to 2.46), based on data from 78 participants in one study	560 per 1000	963 per 1000	Very low (owing to serious risk of bias, serious imprecision, serious indirectness*)	We are uncertain of the effects of proactive therapeutic drug monitoring of adalimumab on sustained disease control or sustained remission
		Difference: 403 more per 1000 (95% CI 112 more to 818 more)			
Sustained remission during 68 weeks	Relative risk 1.72 (95% CI 1.2 to 2.46), based on data from 78 participants in one study	625 per 1000	1000 per 1000	Very low (owing to serious risk of bias, serious imprecision, serious indirectness*)	We are uncertain of the effects of proactive therapeutic drug monitoring of adalimumab on sustained remission
		Difference: 450 more per 1000 (95% CI 125 more to 913 more)			
Remission at 44-68 weeks	Relative risk 1.12 (95% CI 0.86 to 1.46), based on data from 633 participants in three studies	650 per 1000	728 per 1000	Very low (owing to serious imprecision, serious indirectness, serious risk of bias†)	We are uncertain of the effects of proactive therapeutic drug monitoring of adalimumab on remission
		Difference: 78 more per 1000 (95% CI 91 fewer to 299 more)			
Disease worsening, 44 weeks	Relative risk 0.89 (95% CI 0.48 to 1.65), based on data from 218 participants in one study	441 per 1000	392 per 1000	Very low (owing to serious risk of bias, serious imprecision, serious indirectness*)	We are uncertain of the effects of proactive therapeutic drug monitoring of adalimumab on disease worsening
		Difference: 49 fewer per 1000 (95% CI 229 fewer to 287 more)			
Serious adverse events‡, 44-68 weeks	Relative risk 0.76 (95% CI 0.49 to 1.19), based on data from 1053 participants in three studies	80 per 1000	61 per 1000	Very low (owing to very serious imprecision, serious indirectness§)	Proactive therapeutic drug monitoring of adalimumab may have little or no effect on serious adverse events
		Difference: 19 fewer per 1000 (95% CI 41 fewer to 15 more)			
Adverse events leading to disruption of medical treatment, 44-68 weeks	Relative risk 1.07 (95% CI 0.69 to 1.65), based on data from 1053 participants in three studies	50 per 1000	54 per 1000	Very low (owing to very serious imprecision, serious indirectness§)	We are uncertain of the effects of proactive therapeutic drug monitoring of adalimumab on adverse events leading to discontinuation of medical treatment
		Difference: 4 more per 1000 (95% CI 15 fewer to 33 more)			
Anti-drug antibodies, 68 weeks	Relative risk 1.05 (95% CI 0.28 to 3.91), based on data from 78 participants in one study	150 per 1000	158 per 1000	Very low (owing to serious risk of bias, serious imprecision, serious indirectness*)	We are uncertain of the effects of proactive therapeutic drug monitoring of adalimumab on anti-drug antibodies
		Difference: 8 more per 1000 (95% CI 108 fewer to 437 more)			
Sustained disease control	No eligible trials				
Quality of life	No eligible trials				
Change in disease activity	No eligible trials				
Physical function	No eligible trials				
Mental health	No eligible trials				
Work disability	No eligible trials				

* Data come from a trial of patients with Crohn's disease. It is uncertain whether the effects of TDM are consistent across diseases.

† Data come from three trials of patients with inflammatory bowel disease. It is uncertain whether the effects of TDM are consistent across diseases.

‡ One trial first randomised patients with ulcerative colitis during induction to higher versus lower doses of adalimumab.^68^ It subsequently re-randomised patients to adalimumab 40 mg every week, adalimumab 40 mg every other week, or adalimumab with therapeutic drug monitoring. The trial reported efficacy outcomes restricted to patients who achieved remission during induction but reported safety outcomes for the total population regardless of remission status.

§ Data come from three trials of patients with inflammatory bowel disease. It is uncertain whether the effects of TDM are consistent across diseases.

CI, Confidence interval; TDM, Therapeutic drug monitoring.

We are uncertain of the effects of therapeutic drug monitoring of adalimumab on sustained disease control or sustained remission, disease worsening, serious adverse events, and anti-drug antibodies, owing to serious concerns about risk of bias, imprecision, and indirectness. We did not find evidence addressing sustained disease control, change in disease activity, physical function, mental health, quality of life, and work and school disability and absenteeism.

We anticipated that the effect of therapeutic drug monitoring of adalimumab might be different in trials at low versus high risk of bias and based on disease and age. We did not find evidence that the effects of therapeutic drug monitoring of adalimumab were different in trials at low versus high risk of bias or based on disease, although effects across disease subgroups were imprecise (online supplements 5 and 6). Compared with adults, we found that therapeutic drug monitoring of adalimumab might be more effective in children (online supplement 8), although the credibility of the subgroup effect was low according to the ICEMAN tool (online supplement 10).

We did not identify any trials that addressed the effects of proactive therapeutic drug monitoring during maintenance with other biologic drugs. The trial that reported on the effects of proactive therapeutic drug monitoring of infliximab, adalimumab, and etanercept did not report on any of our outcomes of interest.^76^

## Discussion

### Main findings

We present a systematic review and meta-analysis of all randomised trials comparing proactive therapeutic drug monitoring with standard care. For patients undergoing induction, we found low certainty evidence that therapeutic drug monitoring of infliximab may have little or no effect on sustained remission, disease activity, quality of life, physical function, and mental health. Conversely, for patients undergoing maintenance, low certainty evidence suggests that therapeutic drug monitoring of infliximab may increase the proportion of patients who experience sustained disease control or remission, may reduce disease worsening, but may have little or no effect on disease activity, physical function, mental health, and quality of life. The evidence addressing therapeutic drug monitoring of adalimumab during maintenance is very uncertain, although it is compatible with potential benefit.

Our findings have important caveats. Firstly, we did not find evidence relating to the effects of therapeutic drug monitoring of adalimumab during induction or relating to work and school participation. Further, the evidence for safety outcomes such as serious adverse events and adverse events leading to discontinuation of treatment was often at very low certainty.

Duration of follow-up across trials was limited to one year, making the long term effects of proactive therapeutic drug monitoring uncertain. This shortcoming is important because these diseases are chronic, requiring patients to use biologics over extended periods, with flares and disease worsening often occurring after prolonged use of biologics.^77^

We pooled trials investigating therapeutic drug monitoring in inflammatory bowel disease, inflammatory arthritis, and psoriasis on advice of the parallel BMJ Rapid Recommendations guideline panel. The panel followed the GRADE approach, which recommends subgroups to be combined unless compelling evidence indicates any differences.^35^ The panel considered that the best available empirical evidence suggests that the effects of therapeutic drug monitoring are consistent across these immune mediated diseases.^23 24^ This evidence was seen in our systematic review in subgroup analyses, which showed consistent effects of therapeutic drug monitoring across diseases. We acknowledge, however, that the effects from subgroup analyses across diseases are imprecise and that the effect of proactive therapeutic drug monitoring might indeed differ across diseases. Further, most evidence included in this review came from patients with inflammatory bowel disease for which therapeutic drug monitoring might be most effective compared with other diseases, owing to higher rates of anti-drug antibodies. For these reasons, in our application of the GRADE approach to assess the certainty of evidence, we downgraded the certainty of evidence for indirectness.

Likewise, we included evidence from two trials that reported on children.^74 75^ One of these trials addressed the effects of therapeutic drug monitoring of infliximab during maintenance and the other adalimumab during maintenance. The inclusion of these trials reflects extensive deliberations made by the parallel BMJ Rapid Recommendations guideline panel. The panel was cognizant of potential heterogeneity in effects of therapeutic drug monitoring between children and adults but ultimately considered the trial as a source of indirect evidence. Because of these concerns, the certainty of evidence for comparisons in which it was included was downgraded for potential indirectness. Excluding results from the trial of children did not change our findings on therapeutic drug monitoring of infliximab during maintenance. Where the estimates from meta-analyses depended on results from a trial including children (ie, effects of proactive therapeutic drug monitoring of adalimumab during maintenance), the certainty of evidence was downgraded to very low. Ultimately, the consideration of trial evidence including children does not influence the conclusions of this systematic review.

Heterogeneity in the effects of biologics on patient outcomes remains unexplained. For example, it is unclear why some patients respond compared with others who do not, why some patients develop anti-drug antibodies, and why some patients lose response over time despite absence of anti-drug antibodies. Finally, therapeutic drug monitoring was variably implemented across trials. Some trials did not consider anti-drug antibodies at all and target drug levels varied widely across trials. Hence, it is also unclear whether all therapeutic drug monitoring protocols have similar effects.

We did not find any trials addressing therapeutic drug monitoring of biologic drugs other than infliximab, adalimumab, and etanercept, although trials of therapeutic drug monitoring for other biologic drugs are ongoing (NCT03895879). Other trials of therapeutic drug monitoring of infliximab and adalimumab are also ongoing, which could improve the precision of estimates and clarify whether the effects of therapeutic drug monitoring are consistent across immune mediated inflammatory diseases (NCT04775732, NCT02508012, NCT03261102, NCT04835506, ACTRN12621000023853).

### Relation to previous findings

Previous systematic reviews have reported inconsistent results addressing the effects of proactive therapeutic drug monitoring.^31 33^ These reviews, however, were restricted to inflammatory bowel disease and pooled results of trials together across biologic drugs and induction and maintenance. Other observational studies suggest potential benefit of therapeutic drug monitoring, particularly for patients with anti-drug antibodies.^78^,^80^

### Strengths and limitations

The strengths of our review include a comprehensive search strategy, duplicate screening and data collection, and rigorous assessment of the certainty of evidence using the latest GRADE guidance. We also focus on patient important outcomes that were selected by the parallel BMJ Rapid Recommendations guideline panel, including patient partners.

Despite our rigorous search of the literature, it is possible that we missed eligible trials. We mitigated this limitation by also reviewing the references of similar systematic reviews and soliciting experts about eligible trials that might not have come up in our search. We assessed the certainty of evidence using the GRADE approach. While the GRADE approach presents a comprehensive framework for considering all factors that might bear on the certainty of evidence, its application is ultimately subjective, and other readers might come to different conclusions about the certainty of evidence. While we attempted to focus on all outcomes that would be important to patients, none of the trials investigated participation in work or education. We restricted eligibility to randomised trials, which are more likely than non-randomised studies to provide credible estimates on efficacy. Trials, however, could include patients who are less representative of those encountered in clinical practice (eg, punctual and meticulous in attending appointments). Further, only two trials looked at the effects of proactive therapeutic drug monitoring in children and adolescents.^74^ ^75^ Finally, our review focuses on patient important outcomes and we did not review outcomes such as endoscopic remission, because such outcomes might not reflect patients' subjective experience.

### Implications for research and practice

The results of our systematic review and meta-analysis suggest that therapeutic drug monitoring of infliximab during maintenance may be beneficial but that the effects of therapeutic drug monitoring of adalimumab during maintenance or therapeutic drug monitoring during induction are uncertain. Practitioners and other decision makers, however, should be mindful about the cost effectiveness of therapeutic drug monitoring and other additional challenges related to its implementation. While proactive therapeutic drug monitoring has been reported largely cost effective, cost effectiveness analyses have primarily focused on inflammatory bowel disease and North America and western Europe.^81^ The cost effectiveness of therapeutic drug monitoring in other settings and for other diseases is unclear.

There are also challenges with the sensitivity of different assays for measuring drug levels and anti-drug antibodies. The optimal algorithm of modifying drug dosages on the basis of therapeutic drug monitoring is also unclear.^82^ Furthermore, decision makers will need to consider how to offer therapeutic drug monitoring to patients if laboratory facilities and routines for testing and analysing blood samples are not established. Therapeutic drug monitoring of adalimumab will also increase patient burden because it is typically given at home subcutaneously unlike infliximab, which requires patients to receive the drug intravenously with the supervision of healthcare providers at which time serum drug levels can be simultaneously measured.

Critical issues of uncertainty remain that could be resolved in future research. Additional research can investigate whether the effects of therapeutic drug monitoring are consistent across immune mediated inflammatory diseases, optimal protocols for therapeutic drug monitoring, incorporation of pharmacokinetic dashboards, and software decision support tools to assist with therapeutic drug monitoring, and therapeutic drug monitoring of other biologics. It is also unclear whether patients who achieve sustained remission and disease control are better served by continuing or terminating biologic treatment and the optimal sequence of targeted biologic treatments in cases where disease re-emerges.

### Conclusion

Our systematic review and meta-analysis addresses the efficacy and safety of proactive therapeutic drug monitoring in patients with inflammatory bowel disease, inflammatory arthritis, and psoriasis. For patients undergoing induction, we found low certainty evidence that therapeutic drug monitoring of infliximab may have little or no effect on sustained remission, disease activity, quality of life, physical function, and mental health. Conversely, for patients undergoing maintenance, low certainty evidence suggests that proactive therapeutic drug monitoring of infliximab may increase the proportion of patients who experience sustained disease control or remission, may reduce disease worsening, and may have little or no effect on disease activity, physical function, mental health, and quality of life. We are very uncertain of the effects of proactive therapeutic drug monitoring of adalimumab.

## Supplementary material

10.1136/bmjmed-2024-000998 online supplemental file 1

## Data Availability

Data are available in a public, open access repository.
