# Supplementary material for: Proactive therapeutic drug monitoring of biologic drugs in patients with inflammatory bowel disease, inflammatory arthritis, and psoriasis: systematic review and meta-analysis
Source: BMJ Med. 2024 Oct 28;3(1):e000998. doi: 10.1136/bmjmed-2024-000998 (PMC11579540; doi:10.1136/bmjmed-2024-000998)
Supplement: online supplemental file 1 [file bmjmed-3-1-s001.pdf]

Proactive therapeutic drug monitoring of biologic drugs in patients with inflammatory bowel disease, inflammatory arthritis, and psoriasis: A systematic review and meta-analysis

Dena Zeraatkar

zeraatd@mcmaster.ca

Table of Contents

Proactive therapeutic drug monitoring of biologic drugs in patients with inflammatory bowel disease, inflammatory arthritis, and psoriasis: A systematic review and meta-analysis 1

Supplement 1: Search Strategy 4

Supplement 2: Risk of bias guidance 14

Supplement 3: Additional trial and patient characteristics 17

Supplement 4: Forest plots for analyses of therapeutic drug monitoring of infliximab for induction 20

- Remission 20
- Sustained remission 20
- Serious adverse events 20
- Adverse events leading to discontinuation of therapy 20
- Anti-drug antibodies 21

Supplement 5: Subgroup analyses based on risk of bias 22

Supplement 6: Subgroup analyses based on disease 23

Supplement 7: Forest plots for analyses of therapeutic drug monitoring of infliximab for maintenance 29

- Sustained disease control or sustained remission 29
- Sustained disease control 29
- Sustained remission 29
- Remission 29
- Disease worsening 29
- Serious adverse events 30
- Adverse events leading to discontinuation of therapy 30
- Anti-drug antibodies 30

Supplement 8: Subgroup analyses based on age 31

Supplement 9: Forest plots for analyses of therapeutic drug monitoring of adalimumab for maintenance 32

- Sustained disease control or sustained remission 32
- Sustained remission 32
- Remission 32
- Disease worsening 32
- Serious adverse events 32
- Adverse events leading to discontinuation of medical therapy 33
- Anti-drug antibodies 33

Supplement 10: Evaluation of the credibility of the subgroup effect based on age using the ICEMAN tool 34

Supplement 1: Search Strategy
MEDLINE

Database: OVID Medline Epub Ahead of Print, In-Process & Other Non-Indexed Citations, Ovid MEDLINE(R) Daily and Ovid MEDLINE(R) 1946 to Present

#	Searches
1	(drug blood level* or blood level*).mp.
2	(therapeutic drug monitor* or TDM or drug monitor* or proactive monitor*).mp. or exp drug monitoring/
3	(target trough concentration* or trough concentration* or TC* or trough level*).mp.
4	(drug plasma concentration* or drug serum concentration* or drug plasma level* or drug blood concentration* or drug serum level*or plasma concentration* or serum concentration* or plasma level* or blood concentration* or serum level*).mp.
5	antibody titer*.mp.
6	(antibody blood concentration* or antibody plasma concentration* or antibody serum concentration* or antibody blood level* or antibody plasma level* or antibody serum level*).mp.
7	(antibod* to infliximab or ATI or anti-drug antibod* or antidrug antibod* or ADA or anti chimeric or anti-chimeric or antichimeric or human anti-chimeric Ab or HACA).mp.
8	(dashboard driven dos* or point of care test* or POCT).mp.
9	or/1-8
10	(Inflammatory bowel disease or IBD).mp. or exp Inflammatory bowel disease/
11	exp Crohn disease/ or crohn*.mp. or regional ileitis.mp.
12	(ulcerative colitis or colitis).mp. or exp Colitis, Ulcerative/
13	(proctocolitis or proctosigmoiditis or rectocolitis or rectosigmoiditis or proctitis or "distal colitis").mp.
14	exp Lupus Erythematosus, Systemic/
15	arthritis, psoriatic/ or exp arthritis, rheumatoid/
16	polymyositis/ or dermatomyositis/
17	Spondylitis, Ankylosing/
18	(lupus or arthritis or myositis or polymyositis or dermatomyositis or spondylitis).mp. [mp=title, book title, abstract, original title, name of substance word, subject heading word, floating sub-heading word, keyword heading word, organism supplementary concept word, protocol supplementary concept word, rare disease supplementary concept word, unique identifier, synonyms]
19	(chronic adj3 inflammatory).mp. [mp=title, book title, abstract, original title, name of substance word, subject heading word, floating sub-heading word, keyword heading word, organism supplementary concept word, protocol supplementary concept word, rare disease supplementary concept word, unique identifier, synonyms]
20	inflammation.mp. or exp Inflammation/
21	or/10-20
22	9 and 21
23	infliximab.mp. or Infliximab/
24	tumor necrosis factor inhibitors/ or adalimumab/ or certolizumab pegol/ or infliximab/
25	anti TNF.mp.

26	(tumo?r necrosis factor adj3 (inhibit* or block* or antagon*)).mp. [mp=title, book title, abstract, original title, name of substance word, subject heading word, floating sub-heading word, keyword heading word, organism supplementary concept word, protocol supplementary concept word, rare disease supplementary concept word, unique identifier, synonyms]
27	(adalimumab or amlitelimab or belantamab or bleselumab or certolizumab or cudarolimab or denosumab or efizonerimod or efprezimod or etanercept or giloralimab or mitazalimab or pavurutamab or pegilodecakin or pegipanermin or peimine or quisovalimab or ravagalimab or remtolumab or selicrelumab or sotigalimab or tanfanercept or tavolimab or telitacicept or tibulizumab).mp. [mp=title, book title, abstract, original title, name of substance word, subject heading word, floating sub-heading word, keyword heading word, organism supplementary concept word, protocol supplementary concept word, rare disease supplementary concept word, unique identifier, synonyms]
28	or/23-27
29	22 and 28
30	randomized controlled trial.pt.
31	controlled clinical trial.pt.
32	randomi?ed.ab.
33	placebo.ab.
34	drug therapy.fs.
35	randomly.ab.
36	trial.ab.
37	groups.ab.
38	or/30-37
39	exp animals/ not humans.sh.
40	38 not 39
41	29 and 40
42	((IL-17 or IL-6 or IL7 or IL6) adj3 (inhibit* or block or antagon*)).mp. [mp=title, book title, abstract, original title, name of substance word, subject heading word, floating sub-heading word, keyword heading word, organism supplementary concept word, protocol supplementary concept word, rare disease supplementary concept word, unique identifier, synonyms]
43	((interleukin adj3 (inhibit* or block or antagon*)) and (IL-17 or IL-6 or IL7 or IL6)).mp. [mp=title, book title, abstract, original title, name of substance word, subject heading word, floating sub-heading word, keyword heading word, organism supplementary concept word, protocol supplementary concept word, rare disease supplementary concept word, unique identifier, synonyms]
44	(Secukinumab or Ixekizumab or Brodalumab or Bimekizumab or Tocilizumab or Sarilumab or Rituximab or Vendolizumab or Ustekinumab or Abatacept or Risankizumab or Guselkumab or Tildrakizumab or Deucravacitinib).mp. [mp=title, book title, abstract, original title, name of substance word, subject heading word, floating sub-heading word, keyword heading word, organism supplementary concept word, protocol supplementary concept word, rare disease supplementary concept word, unique identifier, synonyms]
45	42 or 43 or 44
46	28 or 45
47	22 and 40 and 46
48	47 not 41
49	limit 41 to ed=20221017-20221208
50	48 or 49

Embase (OVID)

Database: Embase <1974 to 2022 December 07>

#	Searches
1	exp drug blood level/ or (drug blood level* or blood level*).mp.
2	(therapeutic drug monitor* or TDM or drug monitor* or proactive monitor*).mp. or exp drug monitoring/
3	(target trough concentration* or trough concentration* or TC* or trough level*).mp.
4	(drug plasma concentration* or drug serum concentration* or drug plasma level* or drug blood concentration* or drug serum level* or plasma concentration* or serum concentration* or plasma level* or blood concentration* or serum level*).mp.
5	antibody titer*.mp. or exp antibody titer/
6	(antibody blood concentration* or antibody plasma concentration* or antibody serum concentration* or antibody blood level* or antibody plasma level* or antibody serum level*).mp.
7	(antibod* to infliximab or ATI or anti-drug antibod* or antidrug antibod* or ADA or anti chimeric or anti-chimeric or antichimeric or human anti-chimeric Ab or HACA).mp.
8	(dashboard driven dos* or point of care test* or POCT).mp.
9	or/1-8
10	(Inflammatory bowel disease or IBD).mp. or exp Inflammatory bowel disease/
11	exp Crohn disease/ or crohn*.mp. or regional ileitis.mp.
12	(ulcerative colitis or colitis).mp. or exp Colitis, Ulcerative/
13	(proctocolitis or proctosigmoiditis orrectocolitis or rectosigmoiditis or proctitis or "distal colitis").mp.
14	lupus vulgaris/
15	psoriatic arthritis/
16	exp rheumatoid arthritis/
17	polymyositis/
18	dermatomyositis/
19	ankylosing spondylitis/
20	(lupus or arthritis or myositis or polymyositis or dermatomyositis or spondylitis).mp.
21	(chronic adj3 inflammatory).mp.
22	inflammation.mp. or inflammation/
23	or/10-22
24	infliximab.mp. or infliximab/
25	exp tumor necrosis factor inhibitor/
26	anti TNF.mp.
27	(tumo?r necrosis factor adj3 (inhibit* or block* or antagon*)).mp.
28	(adalimumab or amlitelimab or belantamab or bleselumab or certolizumab or cudarolimab or denosumab or efizonerimod or efprezimod or etanercept or giloralimab or mitazalimab or pavurutamab or pegilodecakin or pegipanermin or peimine or quisovalimab or ravagalimab or remtolumab or selicrelumab or sotigalimab or tanfanercept or tavolimab or telitacicept or tibulizumab).mp.

29	or/24-28
30	9 and 23 and 29
31	randomized controlled trial/
32	Controlled clinical study/
33	random$.ti,ab.
34	randomization/
35	intermethod comparison/
36	placebo.ti,ab.
37	(compare or compared or comparison).ti.
38	((evaluated or evaluate or evaluating or assessed or assess) and (compare or compared or comparing or comparison)).ab.
39	(open adj label).ti,ab.
40	((double or single or doubly or singly) adj (blind or blinded or blindly)).ti,ab.
41	double blind procedure/
42	parallel group$1.ti,ab.
43	(crossover or cross over).ti,ab.
44	((assign$ or match or matched or allocation) adj5 (alternate or group$1 or intervention$1 or patient$1 or subject$1 or participant$1)).ti,ab.
45	(assigned or allocated).ti,ab.
46	(controlled adj7 (study or design or trial)).ti,ab.
47	(volunteer or volunteers).ti,ab.
48	human experiment/
49	trial.ti.
50	or/31-49
51	(random$ adj sampl$ adj7 ("cross section$" or questionnaire$1 or survey$ or database$1)).ti,ab. not (comparative study/ or controlled study/ or randomi?ed controlled.ti,ab. or randomly assigned.ti,ab.)
52	Cross-sectional study/ not (randomized controlled trial/ or controlled clinical study/ or controlled study/ or randomi?ed controlled.ti,ab. or control group$1.ti,ab.)
53	(((case adj control$) and random$) not randomi?ed controlled).ti,ab.
54	(Systematic review not (trial or study)).ti.
55	(nonrandom$ not random$).ti,ab.
56	"Random field$".ti,ab.
57	(random cluster adj3 sampl$).ti,ab.
58	(review.ab. and review.pt.) not trial.ti.
59	"we searched".ab. and (review.ti. or review.pt.)
60	"update review".ab.
61	(databases adj4 searched).ab.
62	(rat or rats or mouse or mice or swine or porcine or murine or sheep or lambs or pigs or piglets or rabbit or rabbits or cat or cats or dog or dogs or cattle or bovine or monkey or monkeys or trout or marmoset$1).ti. and animal experiment/
63	Animal experiment/ not (human experiment/ or human/)
64	or/51-63
65	50 not 64
66	30 and 65
67	limit 66 to dc=20211007-20221208

68	((IL-17 or IL-6 or IL7 or IL6) adj3 (inhibit* or block or antagon*)).mp. [mp=title, abstract, heading word, drug trade name, original title, device manufacturer, drug manufacturer, device trade name, keyword heading word, floating subheading word, candidate term word]
69	((interleukin adj3 (inhibit* or block or antagon*)) and (IL-17 or IL-6 or IL7 or IL6)).mp. [mp=title, abstract, heading word, drug trade name, original title, device manufacturer, drug manufacturer, device trade name, keyword heading word, floating subheading word, candidate term word]
70	(Secukinumab or Ixekizumab or Brodalumab or Bimekizumab or Tocilizumab or Sarilumab or Rituximab or Vendolizumab or Ustekinumab or Abatacept or Risankizumab or Guselkumab or Tildrakizumab or Deucravacitinib).mp. [mp=title, abstract, heading word, drug trade name, original title, device manufacturer, drug manufacturer, device trade name, keyword heading word, floating subheading word, candidate term word]
71	68 or 69 or 70
72	71 or 29
73	9 and 23
74	65 and 72 and 73
75	74 not 66
76	67 or 75

Cochrane Library

Search Name: TDM for inflammatory diseases
Date Run: 08/12/2022 17:44:56
Comment:

#	Searches
1	"drug blood level*" or "blood level"
2	"therapeutic drug monitor*" or TDM or "drug monitor*" or "proactive monitor*"
3	MeSH descriptor: [Drug Monitoring] explode all trees
4	"target trough concentration*" or "trough concentration*" or TC* or "trough level*"
5	"drug plasma concentration*" or "drug serum concentration*" or "drug plasma level*" or "drug blood concentration*" or "drug serum level*" or "plasma concentration*" or "serum concentration*" or "plasma level*" or "blood concentration*" or "serum level*"
6	"antibody titer*"
7	"antibody blood concentration*" or "antibody plasma concentration*" or "antibody serum concentration*" or "antibody blood level*" or "antibody plasma level*" or "antibody serum level*"
8	"antibod* to infliximab" or ATI or "anti-drug antibod*" or "antidrug antibod*" or ADA or "anti chimeric" or anti-chimeric or antichimeric or "human anti-chimeric Ab" or HACA
9	"dashboard driven dos*" or "point of care test*" or POCT
10	#1 or #2 or #3 or #4 or #5 or #6 or #7 or #8 or #9
11	MeSH descriptor: [Inflammatory Bowel Diseases] explode all trees
12	"inflammatory bowel disease" or IBD
13	MeSH descriptor: [Crohn Disease] explode all trees
14	crohn* or "regional ileitis"
15	MeSH descriptor: [Colitis, Ulcerative] explode all trees
16	colitis or proctocolitis or proctosigmoiditis or rectocolitis or rectosigmoiditis or proctitis

17	MeSH descriptor: [Lupus Erythematosus, Systemic] explode all trees
18	MeSH descriptor: [Arthritis, Psoriatic] explode all trees
19	MeSH descriptor: [Arthritis, Rheumatoid] explode all trees
20	MeSH descriptor: [Polymyositis] explode all trees
21	MeSH descriptor: [Spondylitis, Ankylosing] explode all trees
22	lupus or arthritis or myositis or polymyositis or dermatomyositis or spondylitis
23	chronic near/3 inflammatory
24	MeSH descriptor: [Inflammation] explode all trees
25	inflammation
26	#11 or #12 or #13 or #14 or #15 or #16 or #17 or #18 or #19 or #20 or #21 or #22 or #23 or #24 or #25
27	#10 and #26
28	MeSH descriptor: [Infliximab] explode all trees
29	infliximab
30	MeSH descriptor: [Tumor Necrosis Factor Inhibitors] explode all trees
31	"anti TNF"
32	tumor necrosis factor near/3 (inhibit* or block* or antagon*)
33	adalimumab or amlitelimab or belantamab or bleselumab or certolizumab or cudarolimab or denosumab or efizonerimod or efprezimod or etanercept or giloralimab or mitazalimab or pavurutamab or pegilodecakin or pegipanermin or peimine or quisovalimab or ravagalimab or remtolumab or selicrelumab or sotigalimab or tanfanercept or tavolimab or telitacicept or tibulizumab
34	#28 or #29 or #30 or #31 or #32 or #33
35	#27 and #34 in Trials
36	((IL-17 or IL-6 or IL7 or IL6) near/3 (inhibit* or block or antagon*))
37	((interleukin near/3 (inhibit* or block or antagon*)) and (IL-17 or IL-6 or IL7 or IL6))
38	Secukinumab or Ixekizumab or Brodalumab or Bimekizumab or Tocilizumab or Sarilumab or Rituximab or Vendolizumab or Ustekinumab or Abatacept or Risankizumab or Guselkumab or Tildrakizumab or Deucravacitinib
39	#36 or #37 or #38
40	#34 or #39
41	#27 and #40 in Trials
42	#41 NOT #35
43	#35 with Cochrane Library publication date in The last 3 months
44	#42 or #43

CINAHL (EBSCO)

#	Query	Limiters/Expanders
S62	S60 OR S61	Search modes - Boolean/Phrase
S61	S56	Limiters - Published Date: 20221001-20221231<br>Search modes - Boolean/Phrase
S60	S59 NOT S56	Search modes - Boolean/Phrase

S59	S49 AND S58	Search modes - Boolean/Phrase
S58	S55 OR S57	Search modes - Boolean/Phrase
S57	TX Secukinumab or Ixekizumab or Brodalumab or Bimekizumab or Tocilizumab or Sarilumab or Rituximab or Vendolizumab or Ustekinumab or Abatacept or Risankizumab or Guselkumab or Tildrakizumab or Deucravacitinib	Search modes - Boolean/Phrase
S56	S49 AND S55	Search modes - Boolean/Phrase
S55	S50 OR S51 OR S52 OR S53 OR S54	Search modes - Boolean/Phrase
S54	TX adalimumab or amlitelimab or belantamab or bleselumab or certolizumab or cudarolimab or denosumab or efizonerimod or efprezimod or etanercept or giloralimab or mitazalimab or pavurutamab or pegilodecakin or pegipanermin or peimine or quisovalimab or ravagalimab or remtolumab or selicrelumab or sotigalimab or tanfanercept or tavolimab or telitacicept or tibulizumab	Search modes - Boolean/Phrase
S53	TX tumor necrosis factor N3 (inhibit* or block* or antagon*)	Search modes - Boolean/Phrase
S52	"anti TNF"	Search modes - Boolean/Phrase
S51	(MH "Tumor Necrosis Factor Inhibitors")	Search modes - Boolean/Phrase
S50	"infliximab"	Search modes - Boolean/Phrase
S49	S23 AND S34 AND S48	Search modes - Boolean/Phrase
S48	S35 OR S36 OR S37 OR S38 OR S39 OR S40 OR S41 OR S42 OR S43 OR S44 OR S45 OR S46 OR S47	Search modes - Boolean/Phrase
S47	(MH "Inflammation") OR "inflammation"	Search modes - Boolean/Phrase
S46	TX chronic N3 inflammatory	Search modes - Boolean/Phrase
S45	TX lupus or arthritis or myositis or polymyositis or dermatomyositis or spondylitis	Search modes - Boolean/Phrase
S44	(MH "Spondylitis, Ankylosing")	Search modes - Boolean/Phrase
S43	(MH "Polymyositis") OR (MH "Dermatomyositis")	Search modes - Boolean/Phrase
S42	(MH "Arthritis, Psoriatic") OR (MH "Arthritis, Rheumatoid+")	Search modes - Boolean/Phrase
S41	(MH "Lupus Erythematosus, Systemic")	Search modes - Boolean/Phrase

S40	TX colitis or proctocolitis or proctosigmoiditis or rectocolitis or rectosigmoiditis or proctitis	Search modes - Boolean/Phrase
S39	(MH "Colitis, Ulcerative")	Search modes - Boolean/Phrase
S38	TX crohn* or regional ileitis	Search modes - Boolean/Phrase
S37	(MH "Crohn Disease")	Search modes - Boolean/Phrase
S36	TX Inflammatory bowel disease or IBD	Search modes - Boolean/Phrase
S35	(MH "Inflammatory Bowel Diseases+")	Search modes - Boolean/Phrase
S34	S24 OR S25 OR S26 OR S27 OR S28 OR S29 OR S30 OR S31 OR S32 OR S33	Search modes - Boolean/Phrase
S33	TX dashboard driven dos* or point of care test* or POCT	Search modes - Boolean/Phrase
S32	TX antibod* to infliximab or ATI or anti-drug antibod* or antidrug antibod* or ADA or anti chimeric or anti-chimeric or antichimeric or human anti-chimeric Ab or HACA	Search modes - Boolean/Phrase
S31	TX antibody blood concentration* or antibody plasma concentration* or antibody serum concentration* or antibody blood level* or antibody plasma level* or antibody serum level*	Search modes - Boolean/Phrase
S30	"antibody titer*"	Search modes - Boolean/Phrase
S29	TX drug plasma concentration* or drug serum concentration* or drug plasma level* or drug blood concentration* or drug serum level*or plasma concentration* or serum concentration* or plasma level* or blood concentration* or serum level*	Search modes - Boolean/Phrase
S28	TX target trough concentration* or trough concentration* or TC* or trough level*	Search modes - Boolean/Phrase
S27	TX therapeutic drug monitor* or TDM or drug monitor* or proactive monitor*	Search modes - Boolean/Phrase
S26	(MH "Drug Monitoring")	Search modes - Boolean/Phrase
S25	"blood level"	Search modes - Boolean/Phrase
S24	"drug blood level"	Search modes - Boolean/Phrase
S23	S22 NOT S21	Expanders - Apply equivalent subjects<br>Search modes - Boolean/Phrase
S22	S1 OR S2 OR S3 OR S4 OR S5 OR S6 OR S7 OR S8 OR S9 OR S10 OR S11 OR S12 OR S13 OR S14 OR S15	Expanders - Apply equivalent subjects<br>Search modes - Boolean/Phrase

S21	S19 NOT S20	Expanders - Apply equivalent subjects<br>Search modes - Boolean/Phrase
S20	MH (human)	Expanders - Apply equivalent subjects<br>Search modes - Boolean/Phrase
S19	S16 OR S17 OR S18	Expanders - Apply equivalent subjects<br>Search modes - Boolean/Phrase
S18	TI (animal model*)	Expanders - Apply equivalent subjects<br>Search modes - Boolean/Phrase
S17	MH (animal studies)	Expanders - Apply equivalent subjects<br>Search modes - Boolean/Phrase
S16	MH animals+	Expanders - Apply equivalent subjects<br>Search modes - Boolean/Phrase
S15	AB (cluster W3 RCT)	Expanders - Apply equivalent subjects<br>Search modes - Boolean/Phrase
S14	MH (crossover design) OR MH (comparative studies)	Expanders - Apply equivalent subjects<br>Search modes - Boolean/Phrase
S13	AB (control W5 group)	Expanders - Apply equivalent subjects<br>Search modes - Boolean/Phrase
S12	PT (randomized controlled trial)	Expanders - Apply equivalent subjects<br>Search modes - Boolean/Phrase
S11	MH (placebos)	Expanders - Apply equivalent subjects<br>Search modes - Boolean/Phrase
S10	MH (sample size) AND AB (assigned OR allocated OR control)	Expanders - Apply equivalent subjects<br>Search modes - Boolean/Phrase

S9	TI (trial)	Expanders - Apply equivalent subjects<br>Search modes - Boolean/Phrase
S8	AB (random*)	Expanders - Apply equivalent subjects<br>Search modes - Boolean/Phrase
S7	TI (randomised OR randomized)	Expanders - Apply equivalent subjects<br>Search modes - Boolean/Phrase
S6	MH cluster sample	Expanders - Apply equivalent subjects<br>Search modes - Boolean/Phrase
S5	MH pretest-posttest design	Expanders - Apply equivalent subjects<br>Search modes - Boolean/Phrase
S4	MH random assignment	Expanders - Apply equivalent subjects<br>Search modes - Boolean/Phrase
S3	MH single-blind studies	Expanders - Apply equivalent subjects<br>Search modes - Boolean/Phrase
S2	MH double-blind studies	Expanders - Apply equivalent subjects<br>Search modes - Boolean/Phrase
S1	MH randomized controlled trials	Expanders - Apply equivalent subjects<br>Search modes - Boolean/Phrase

Supplement 2: Risk of bias guidance

**Bias from the randomization process**	
Issues to consider:<br>Random sequence generation<br>Allocation concealment	
**Definitely low risk of bias**	Trials that assign participants to alternative interventions using a randomly generated sequence and maintain allocation concealment.<br><br>Examples of methods for developing a randomly generated allocation sequence include a random number generator, random number table, coin tossing, shuffling cards or envelopes, and throwing dice. If a trial is described as 'randomized' without any additional details related to how the allocation sequence was developed, we will assume that the allocation sequence was appropriately developed.<br><br>Examples of methods for maintaining allocation concealment include using central allocation via a computer or phone system, pharmacy-controlled allocation, opaque sealed envelopes, and sequentially numbered drug containers.<br><br>*Note that an explicit description of random sequence generation is not necessary for a rating of low risk of bias.*
**Probably low risk of bias**	Trials in which healthcare providers were blind to the intervention but which provide no information on allocation concealment.<br><br>*Note that an explicit description of random sequence generation is not necessary for a rating of probably low risk of bias.*
**Probably high risk of bias**	Trials in which healthcare providers were not blind to the intervention and which provide no information on allocation concealment.<br><br>Trials in which there are substantial baseline differences between trial arms that suggest a problem with the randomization process but there are no other limitations related to randomization.
**Definitely high risk of bias**	Trials in which allocation is by judgment of the clinician, by preference of the participant, by availability of the intervention, based on the results of a laboratory test, or other non-random rules (e.g., birthdate, etc.).<br><br>Trials in which investigators enrolling participants could possibly foresee the arm to which each subsequent patient would be randomized, such as allocation using an open allocation schedule (e.g. a list of random numbers), assignment envelopes used without appropriate safeguards (e.g. use of unsealed, non-opaque or not sequentially numbered envelopes), alternation between arms, case record number, or any other explicitly unconcealed procedure, rate as high risk.
**Bias due to deviations from the intended intervention**	
Issues to consider:	

Blinding of healthcare providers/clinicians and participants<br>Imbalances in cointerventions or behaviors	
**Definitely low risk of bias**	Trials in which healthcare providers are blind to the intervention administered and in which there are no significant differences in administered co-interventions.<br><br>Trials that are described as double or triple blind.
**Probably low risk of bias**	
**Probably high risk of bias**	Trials in which healthcare providers are not blind to the intervention administered.<br><br>Trials in which healthcare providers are blind to the intervention administered but there are significant differences in administered co-interventions that suggests that blinding may have been compromised.<br><br>Trials in which healthcare providers are described as being blind to the intervention but allocation concealment was inadequate.
**Definitely high risk of bias**	Trials in which healthcare providers are not blind to the intervention and in which there are significant differences in administered co-interventions.
**Bias due to missing data**	
Issues to consider:<br>Missing outcome measures<br>Loss to follow-up	
**Definitely low risk of bias**	Trials in which missing outcome data (including outcome data that has been imputed) < 10%.
**Probably low risk of bias**	Trials in which missing outcome data (including outcome data that has been imputed) is between 10% to 15% and missing outcome data is unlikely to be related to the true outcome and there is no imbalance in numbers of or reasons for missing data across intervention groups.
**Probably high risk of bias**	Trials in which missing outcome data (including outcome data that has been imputed) is between 10% to 15% and missing outcome data is likely to be related to the true outcome or there are imbalances in numbers of or reasons for missing data across intervention groups.
**Definitely high risk of bias**	Trials in which missing outcome data (including outcome data that has been imputed) > 15%.
**Bias due to measurement of the outcome**	
Issues to consider:<br>Blinding of outcome adjudicators<br>Objectivity of outcome<br><br>*Note that the judgments may differ across outcomes.*	
**Definitely low risk of bias**	Trials in which patients are blind to the intervention and in which outcomes are patient-reported.<br><br>Trials in which outcomes are measured by a third-party (investigator or clinician) and in which the third-party is blind to the intervention.

	Trials in which the outcomes are objective (e.g., mortality, hospitalization).<br><br>Trials that are described as double or triple blind.
**Probably low risk of bias**	
**Probably high risk of bias**	
**Definitely high risk of bias**	Trials in which patients are not blind and in which outcomes are patient-reported (e.g., quality of life).<br><br>Trials in which outcome adjudicators are not blind and the outcomes are not objective (e.g., adverse events leading to discontinuation).
**Bias in selection of the reported results**	
Issues to consider:<br>Selective reporting of timepoints<br>Selective reporting of outcome measures<br><br>*Note that we are only interested in selective reporting for the outcomes for which we are extracting data.*<br><br>*Note that the judgments may differ across outcomes.*	
**Definitely low risk of bias**	Results for outcomes that were analyzed and reported according to a pre-specified statistical analysis plan or protocol (including the timepoint for the measurement of the outcome).
**Probably low risk of bias**	Results for outcomes that were analyzed and reported but that were not prespecified in a statistical analysis plan or protocol but the timepoint at which results are reported is consistent with the timepoint for other outcomes in the trial report or there is little reason to believe the outcome was selectively reported.<br><br>Please note that outcomes that were not prespecified in a protocol or statistical analysis plan and that are reported in the trial preprint or publication should be rated at probably low risk of bias unless there are other important reasons to suspect that results for those outcomes were selectively reported (e.g., results are presented at timepoints that don't match the timepoints reported for other outcomes).
**Probably high risk of bias**	Results for outcomes that were analyzed and reported but that were not prespecified in a statistical analysis plan or protocol but the timepoint at which results are reported is not consistent with the timepoint for other outcomes in the trial report or there are other reasons to believe that the outcome is selectively reported.
**Definitely high risk of bias**	Results for outcomes that were analyzed and reported for which there are inconsistencies with the statistical analysis plan or protocol. These inconsistencies may include outcome measures of interest or the timepoints for the measurement of outcomes.

Supplement 3: Additional trial and patient characteristics

Trial	Drug	Initial dose	Frequency of TDM	Drug threshold	Adjustment in case of subtherapeutic drug concentration	Range of dose allowed	Range of interval allowed	Access to anti-drug antibody measures	Antibody threshold for halting	Steroids	Using steroids (%)	Concomitant immunosuppressive therapy (%)	Previous use of biologic therapy (%)	Comparator regimen
							**Induction**							
**D'Haens, 2018 (TAILORIX)**	infliximab	5 mg/kg at weeks 0, 2, and 6	Weeks 2, 6, 14, 22, 30, 38, 46, and 54	3 ug/mL to 10 ug/mL	dose	5 mg/kg to 10 mg/kg	2 to 4 weeks	Available at the end of study only	NA	Tapered, low dose immunomodulators allowed.	NR	100	0	5 mg/kg at weeks 0, 2, and 6 , Dose increase by 5 to 10 mg/kg if patients had a CDAI >220 at the current visit or a CDAI between 150 and 220
**Syversen, 2021 (NOR-DRUM-A)**	infliximab	5mg/kg (3mg/kg for rheumatoid arthritis) at 0, 2, and 6 weeks and every eighth week thereafter	At each infusion	3 to 8 mg/L	dose, interval	2.5 mg/kg to 10 mg/kg	4 to 10 weeks	Yes	50 ug/L ≤	NA	17.8	55.5	23.1	Adjustment according to clinical parameters
							**Maintenance**							
**Assa, 2019 (PAILOT)**	adalimumab	Every 2 weeks, either 40 mg in children weighing ≥40 kg or 25 mg/m2 body surface area in children <40 kg	Every 8 weeks (physicians informed within 2 weeks of sampling)	5 to 10 ug/mL	interval	No dose adaption	1 to 2 weeks	Yes	8 ug/L ≤	Maximal dose 1.0– 1.5 mg/kg up to 40 mg for 2 weeks, tapering off by 5 mg every week until completion	NR	43.5	0	Every 2 weeks, either 40 mg in children weighing ≥40 kg or 25 mg/m2 body surface area in children <40 kg
**Casteele, 2015 (TAXIT)**	infliximab	5 mg/kg but allowed to vary before study start	At each infusion	3 to 7 ug/mL	dose, interval	5 to 10 mg/kg	4 to 12 weeks	Yes	8 ug/L ≤	Allowed concomitant low dose immunomodulators/steroids	NR	5.2	NR	Dosing based on clinical symptoms and C-reactive protein (CRP)
**D'Haens, 2022 (SERENE CD)**	adalimumab	For HIR, patients received adalimumab 160 mg at baseline, and at week 1, week 2, and week 3. For SIR, patients received adalimumab	Weeks 12, 26, 40, and 56	5 ug/mL to 20 ug/mL	interval	NA	1 to 2 weeks	Yes	NR	Maximal dose 40 mg/day, tapering off weekly by 5 mg/day prednisone (or equivalent) for doses > 10 mg/day of prednisone (or equivalent) until a 10 mg/day (or equivalent) dose	51.6	30.4	0	40 mg every other week, escalated to 40 mg ew if the patient's CDAI score was ≥220 or hs-CRP level was ≥10 mg/ L

		160 mg at baseline, placebo (adalimumab vehicle) at week 1, adalimumab 80 mg at week 2, and placebo at week 3. Starting at week 4, patients in both groups received adalimumab 40 mg eow through week 12								was reached, then a weekly decrease by 2.5 mg/day (or equivalent) until discontinuation.				
**Kang, 2024**	Infliximab	NR	NR	NR	NR	NR	NR	NR	NR	NR	NR	NR	0	Clinically based dosing
**Panes, 2022 (SERENE UC)**	adalimumab	In the induction study, patients were randomized 3:2 using an interactive response system to receive the higher induction regimen of 160 mg at weeks 0, 1, 2, and 3, followed by 40 mg at weeks 4 and 6 or the standard induction regimen (SIR) of adalimumab 160 mg at week 0, followed by adalimumab 80 mg at week 2 and 40 mg at weeks 4 and 6 (placebo received at weeks 1 and 3 to maintain blinding)	Weeks 2, 4, 8, 10, 12, 16, 22, 24, 29, 35, 37, 42, 48, and 52 (physician informed in 2 weeks)	10 to 20 mg/mL	dose, interval	The dose for patients receiving 40 mg every other week who met dose adjustment criteria was escalated to 40 mg every week; The dose for patients receiving 40 mg ew who met the dose adjustment criteria received a one-time dose of 160 mg at the visit, and resumed 40 mg ew starting the following week.	1 to 2 weeks	Yes	20 ug/L ≤	Maximal dose 40 mg/day	61.2	25.6	13.3	Treatment without dose adaptations
**Pfeiffer-Jensen, 2022**	infliximab	NA	Weeks 12, 24, 36 and 48	1.5 to 6.5 mg/L	Treatment was paused when serum trough	NR	NR	No	NR	NR	NR	NR	NR	Treatment without dose adaptations

					levels were lower than the lower limit in the therapeutic interval									
**Pfeiffer-Jensen, 2022**	etanercept	NA	Weeks 12, 24, 36 and 48	> 1.5 mg/L	Treatment was paused when serum trough levels were lower than the lower limit in the therapeutic interval	25 to 50 mg	6 to 9 days	No	NR	NR	NR	40	NR	Treatment without dose adaptations
**Pfeiffer-Jensen, 2022**	adalimumab	NA	Weeks 12, 24, 36 and 48	5 to 8 mg/L	Treatment was paused when serum trough levels were lower than the lower limit in the therapeutic interval	NR	14 to 18 days	No	NR	NR	NR	40	NR	Treatment without dose adaptations
**Strik, 2020 (PRECISION)**	infliximab	NA	At each infusion	3 ug/mL	dose, interval	1 to 10 mg/mg	4 to 12 weeks	Yes	NR	Allowed concomitant low dose immunomodulators/steroids	NR	40	NR	Treatment without dose adaptations
**Syversen, 2021 (NOR-DRUM-B)**	infliximab	NA	At each infusion	3 to 8 mg/L	dose, interval	2 to 10 mg/kg	4 to 10 weeks	Yes	50 ug/L ≤	Allowed concomitant immunomodulators/steroids	17.8	55.7	27.5	Adjustment according to clinical judgement

Supplement 4: Forest plots for analyses of therapeutic drug monitoring of infliximab for induction

Remission

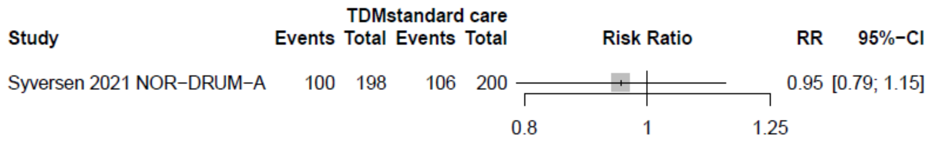

Sustained remission

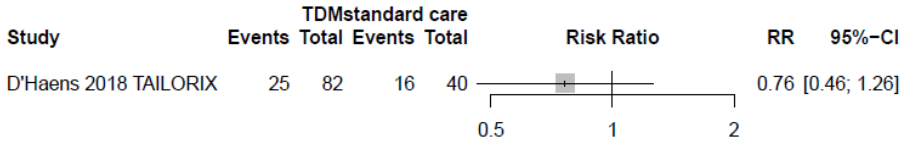

Serious adverse events

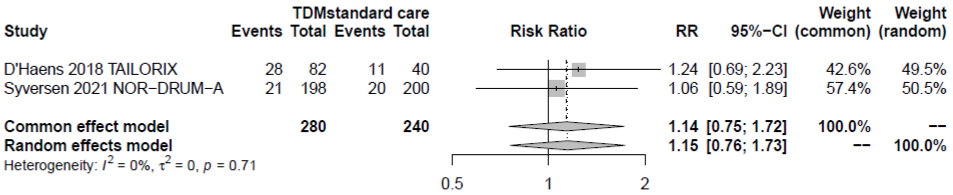

Adverse events leading to discontinuation of therapy

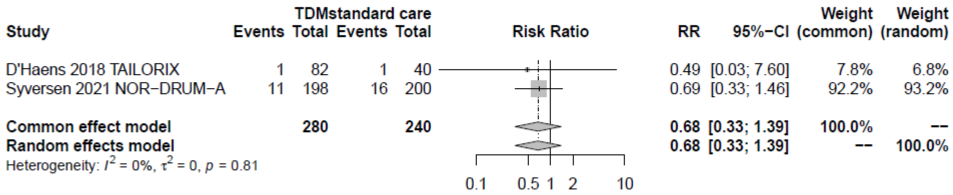

Anti-drug antibodies

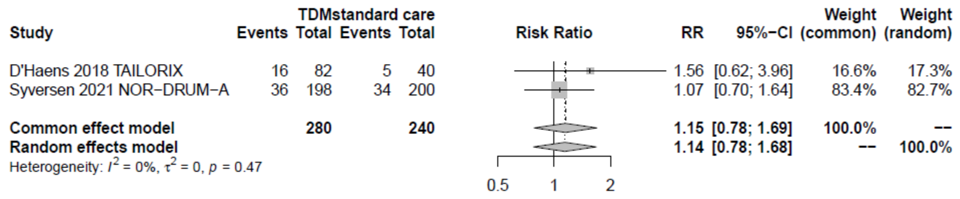

Supplement 5: Subgroup analyses based on risk of bias

Patient group	Drug	Outcome	Subgroup	Studies	Patients	RR (95% CI)	I2	P value for interaction
**Induction**	Infliximab	Serious adverse events	high	1	398	1.06 (0.59, 1.89)	NA	0.71
			low	1	122	1.24 (0.69, 2.23)	NA	
		Adverse events leading to discontinuation of therapy	high	1	398	0.69 (0.33, 1.46)	NA	0.81
			low	1	122	0.49 (0.03, 7.6)	NA	
		Anti-drug antibodies	low	1	122	1.56 (0.62, 3.96)	NA	0.47
			high	1	398	1.07 (0.70, 1.64)	NA	
**Maintenance**	infliximab	Sustained disease control or remission	high	3	646	1.27 (1.15, 1.42)	0	0.37
			low	1	226	1.03 (0.66, 1.61)	NA	
		Remission	high	1	437	1.02 (0.89, 1.17)	NA	0.84
			low	1	251	1.04 (0.88, 1.24)	NA	
		Sustained remission	high	2	192	1.22 (1.04, 1.44)	0	0.49
			low	1	226	1.03 (0.66, 1.61)	NA	
		Serious adverse events	high	2	534	0.93 (0.49, 1.76)	0	0.5
			low	1	251	2.88 (0.12, 70.1)	NA	
		Anti-drug antibody	high	1	408	0.61 (0.36, 1.05)	NA	0.33
			low	1	251	0.14 (0.01, 2.63)	NA	
	Adalimumab	Remission	high	2	262	1.15 (0.75, 1.76)	83.6	0.77
			low	1	371	1.06 (0.76, 1.49)	NA	
		Serious adverse events	high	1	78	1.05 (0.28, 3.91)	NA	0.61
			low	2	975	0.73 (0.46, 1.17)	0	
		Adverse events leading to discontinuation of therapy	high	1	78	5.26 (0.26, 106.08)	NA	0.29
			low	2	975	1.03 (0.66, 1.6)	0	

Supplement 6: Subgroup analyses based on disease

Patient group	Drug	Outcome	Subgroup	Studies	Participants	RR (95% CI)	I2	P value for interaction
**Induction**	Infliximab	Remission	psoriasis	1	22	1.04 (0.58, 1.87)	NA	0.58
			psoriatic arthritis	1	42	0.46 (0.2, 1.07)	NA	
			crohn's disease	1	57	0.97 (0.63, 1.48)	NA	
			ulcerative colitis	1	80	0.91 (0.67, 1.23)	NA	
			rheumatoid arthritis	1	80	1.11 (0.73, 1.68)	NA	
			spondylarthritis	1	117	1.08 (0.67, 1.72)	NA	
**Maintenance**	Infliximab	Remission	ulcerative colitis	2	142	0.95 (0.81, 1.12)	0	0.48
			crohn's disease	2	250	1.16 (0.97, 1.39)	0	
			rheumatoid arthritis	1	77	1.04 (0.81, 1.35)	NA	
			spondylarthritis	1	134	1 (0.71, 1.41)	NA	
			psoriasis	1	33	1.09 (0.81, 1.45)	NA	
			psoriatic arthritis	1	52	0.81 (0.58, 1.15)	NA	
		Sustained remission	crohn's disease	2	178	1.31 (1.12 to 1.54)	0	0.76
			ulcerative colitis	1	14	1.2 (0.69, 2.1)	NA	
		Sustained disease control	psoriasis	1	37	1.36 (0.86, 2.14)	NA	0.9
			crohn's disease	1	66	1.33 (0.9, 1.96)	NA	
			spondylarthritis	1	138	1.36 (1.08, 1.71)	NA	
			ulcerative colitis	1	81	1.45 (1.01, 2.1)	NA	
			psoriatic arthritis	1	53	1.06 (0.72, 1.56)	NA	
			rheumatoid arthritis	1	79	1.26 (0.89, 1.79)	NA	

	Adalimumab	Remission	crohn's disease	2	262	1.15 (0.75, 1.76)	83.6	0.77
			ulcerative colitis	1	371	1.06 (0.76, 1.49)	NA	
		Serious adverse events	crohn's disease	2	296	0.84 (0.36, 1.97)	0	0.79
			ulcerative colitis	1	757	0.73 (0.44, 1.24)	NA	
		Adverse events leading to discontinuation of therapy	crohn's disease	2	294	1.26 (0.53, 3.02)	0	0.66
			ulcerative colitis	1	757	1 (0.61, 1.66)	NA	

Subgroup analysis of the outcome remission based on disease for the analysis of infliximab during induction

Study	Experimental Events	Experimental Total	Control Events	Control Total	RR	95%-CI	Weight (common)	Weight (random)
subgroup = ulcerative colitis								
Syversen 2021 NOR-DRUM-A	25	39	29	41	0.91	[0.67; 1.23]	26.9%	34.6%
subgroup = psoriasis								
Syversen 2021 NOR-DRUM-A	9	13	6	9	1.04	[0.58; 1.87]	6.7%	9.4%
subgroup = psoriatic arthritis								
Syversen 2021 NOR-DRUM-A	5	20	12	22	0.46	[0.20; 1.07]	10.9%	4.5%
subgroup = crohn's disease								
Syversen 2021 NOR-DRUM-A	17	29	17	28	0.97	[0.63; 1.48]	16.4%	17.8%
subgroup = rheumatoid arthritis								
Syversen 2021 NOR-DRUM-A	21	38	21	42	1.11	[0.73; 1.68]	19.0%	18.8%
subgroup = spondyloarthritis								
Syversen 2021 NOR-DRUM-A	23	59	21	58	1.08	[0.67; 1.72]	20.1%	14.9%
**Common effect model**		**198**		**200**	**0.95**	**[0.79; 1.14]**	**100.0%**	**--**
**Random effects model**					**0.96**	**[0.80; 1.15]**	**--**	**100.0%**

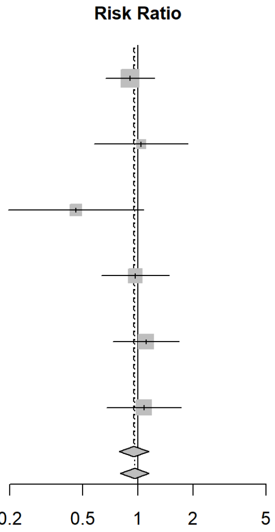

Heterogeneity: $I^2 = 0\%$, $\tau^2 < 0.0001$, $p = 0.58$
Test for subgroup differences (common effect): $\chi^2_5 = 3.79$, df = 5 ($p = 0.58$)
Test for subgroup differences (random effects): $\chi^2_5 = 3.79$, df = 5 ($p = 0.58$)

Subgroup analysis of the outcome remission based on disease for the analysis of infliximab during maintenance

Study	Experimental Events	Experimental Total	Control Events	Control Total	RR	95%-CI	Weight (common)	Weight (random)
subgroup = crohn's disease								
Casteele 2015 TAXIT	57	91	45	82	1.14	[0.89; 1.47]	21.1%	13.9%
Syversen 2021 NOR-DRUM-B	29	36	28	41	1.18	[0.91; 1.53]	11.6%	12.8%
Common effect model		**127**		**123**	**1.15**	**[0.96; 1.39]**	**32.7%**	--
Random effects model					**1.16**	**[0.97; 1.39]**	--	**26.7%**
Heterogeneity: $I^2 = 0\%$, $\tau^2 = 0$, $p = 0.86$								
subgroup = ulcerative colitis								
Casteele 2015 TAXIT	31	37	36	41	0.95	[0.80; 1.14]	15.2%	26.8%
Syversen 2021 NOR-DRUM-B	21	32	22	32	0.95	[0.68; 1.34]	9.8%	7.6%
Common effect model		**69**		**73**	**0.95**	**[0.80; 1.14]**	**25.0%**	--
Random effects model					**0.95**	**[0.81; 1.12]**	--	**34.4%**
Heterogeneity: $I^2 = 0\%$, $\tau^2 = 0$, $p = 1.00$								
subgroup = spondyloarthritis								
Syversen 2021 NOR-DRUM-B	32	65	34	69	1.00	[0.71; 1.41]	14.7%	7.5%
subgroup = psoriasis								
Syversen 2021 NOR-DRUM-B	15	17	13	16	1.09	[0.81; 1.45]	6.0%	10.4%
subgroup = psoriatic arthritis								
Syversen 2021 NOR-DRUM-B	18	28	19	24	0.81	[0.58; 1.15]	9.1%	7.5%
subgroup = rheumatoid arthritis								
Syversen 2021 NOR-DRUM-B	30	39	28	38	1.04	[0.81; 1.35]	12.6%	13.5%
**Common effect model**		**345**		**343**	**1.03**	**[0.93; 1.14]**	**100.0%**	--
**Random effects model**					**1.02**	**[0.93; 1.12]**	--	**100.0%**

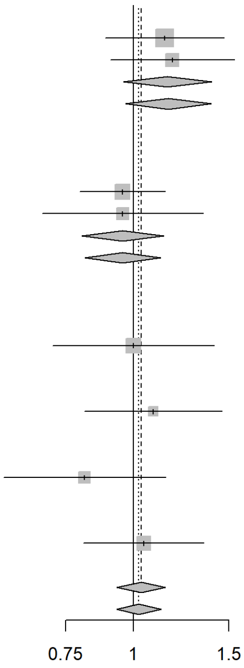

Heterogeneity: $I^2 = 0\%$, $\tau^2 = 0$, $p = 0.72$
Test for subgroup differences (common effect): $\chi^2_5 = 4.16$, df = 5 ($p = 0.53$)
Test for subgroup differences (random effects): $\chi^2_5 = 4.47$, df = 5 ($p = 0.48$)

Subgroup analysis of the outcome sustained remission based on disease for the analysis of infliximab during maintenance

Study	Experimental Events	Experimental Total	Control Events	Control Total	RR	95%-CI	Weight (common)	Weight (random)
subgroup = crohn's disease								
Kang 2023 NR	51	57	39	55	1.26	[1.04; 1.53]	60.4%	63.1%
Strik 2020 PRECISION	30	33	21	33	1.43	[1.08; 1.89]	32.0%	29.5%
Common effect model		**90**		**88**	**1.32**	**[1.13; 1.55]**	**92.4%**	--
Random effects model					**1.31**	**[1.12; 1.54]**	--	**92.6%**
Heterogeneity: $I^2 = 0\%$, $\tau^2 = 0$, $p = 0.47$								
subgroup = ulcerative colitis								
Strik 2020 PRECISION	6	7	5	7	1.20	[0.69; 2.10]	7.6%	7.4%
**Common effect model**		**97**		**95**	**1.31**	**[1.13; 1.53]**	**100.0%**	--
**Random effects model**					**1.30**	**[1.12; 1.52]**	--	**100.0%**

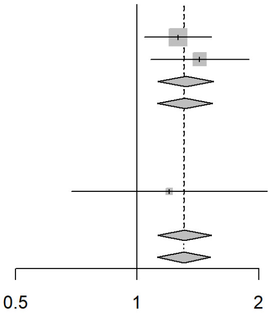

Heterogeneity: $I^2 = 0\%$, $\tau^2 = 0$, $p = 0.74$
Test for subgroup differences (common effect): $\chi^2_1 = 0.10$, df = 1 ($p = 0.75$)
Test for subgroup differences (random effects): $\chi^2_1 = 0.09$, df = 1 ($p = 0.76$)

Subgroup analysis of the outcome sustained disease control based on disease for the analysis of infliximab during maintenance

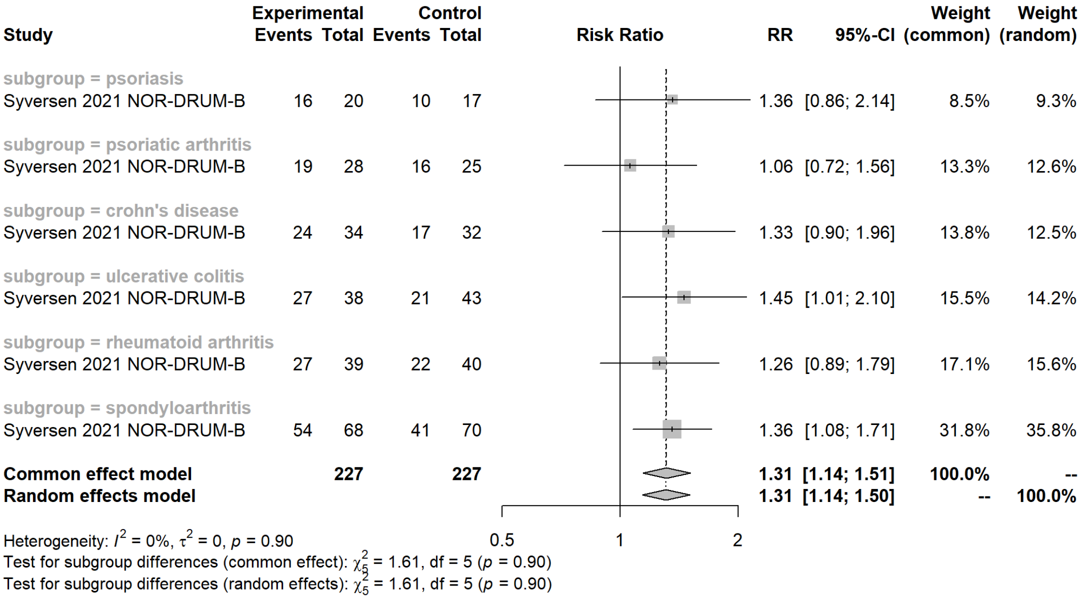

Subgroup analysis of the outcome remission based on disease for the analysis of adalimumab during maintenance

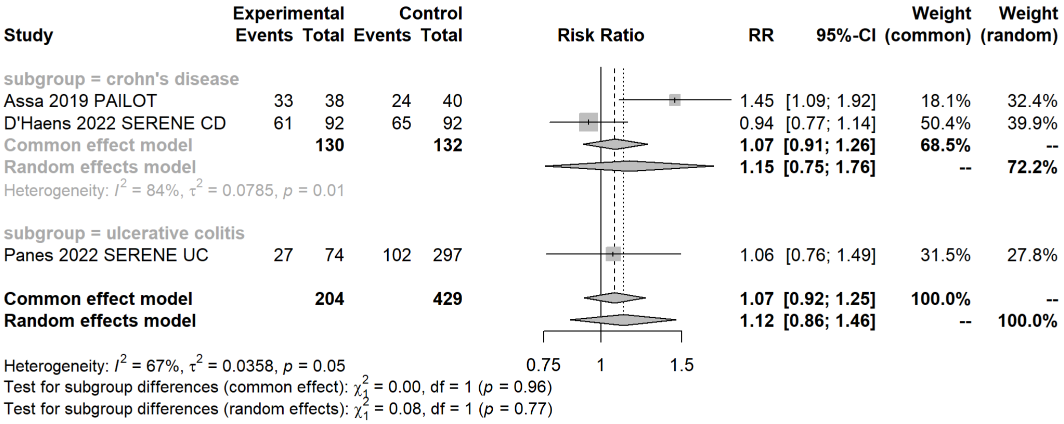

Subgroup analysis of the outcome serious adverse events based on disease for the analysis of adalimumab during maintenance

Study	Experimental Events	Experimental Total	Control Events	Control Total	RR	95%-CI	Weight (common)	Weight (random)
subgroup = crohn's disease								
Assa 2019 PAILOT	4	38	4	40	1.05	[0.28; 3.91]	8.9%	11.4%
D'Haens 2022 SERENE CD	5	109	7	109	0.71	[0.23; 2.18]	16.1%	15.8%
**Common effect model**		**147**		**149**	**0.84**	**[0.36; 1.95]**	**25.0%**	**--**
**Random effects model**					**0.84**	**[0.36; 1.97]**	**--**	**27.3%**
Heterogeneity: $I^2 = 0\%$, $\tau^2 = 0$, $p = 0.66$								
subgroup = ulcerative colitis								
Panes 2022 SERENE UC	15	151	82	606	0.73	[0.44; 1.24]	75.0%	72.7%
**Common effect model**		**298**		**755**	**0.76**	**[0.49; 1.18]**	**100.0%**	**--**
**Random effects model**					**0.76**	**[0.49; 1.19]**	**--**	**100.0%**

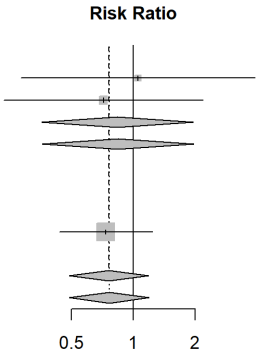

Heterogeneity: $I^2 = 0\%$, $\tau^2 = 0$, $p = 0.88$
Test for subgroup differences (common effect): $\chi^2_1 = 0.06$, df = 1 ($p = 0.80$)
Test for subgroup differences (random effects): $\chi^2_1 = 0.07$, df = 1 ($p = 0.79$)

Subgroup analysis of the outcome adverse events leading to discontinuation of therapy based on disease for the analysis of adalimumab during maintenance

Study	Experimental Events	Experimental Total	Control Events	Control Total	RR	95%-CI	Weight (common)	Weight (random)
subgroup = crohn's disease								
Assa 2019 PAILOT	2	38	0	40	5.26	[0.26; 106.08]	1.4%	2.1%
D'Haens 2022 SERENE CD	9	109	8	107	1.10	[0.44; 2.76]	22.6%	22.6%
**Common effect model**		**147**		**147**	**1.34**	**[0.57; 3.15]**	**24.0%**	**--**
**Random effects model**					**1.26**	**[0.53; 3.02]**	**--**	**24.7%**
Heterogeneity: $I^2 = 0\%$, $\tau^2 = 0$, $p = 0.33$								
subgroup = ulcerative colitis								
Panes 2022 SERENE UC	17	151	68	606	1.00	[0.61; 1.66]	76.0%	75.3%
**Common effect model**		**298**		**753**	**1.08**	**[0.70; 1.67]**	**100.0%**	**--**
**Random effects model**					**1.06**	**[0.69; 1.64]**	**--**	**100.0%**

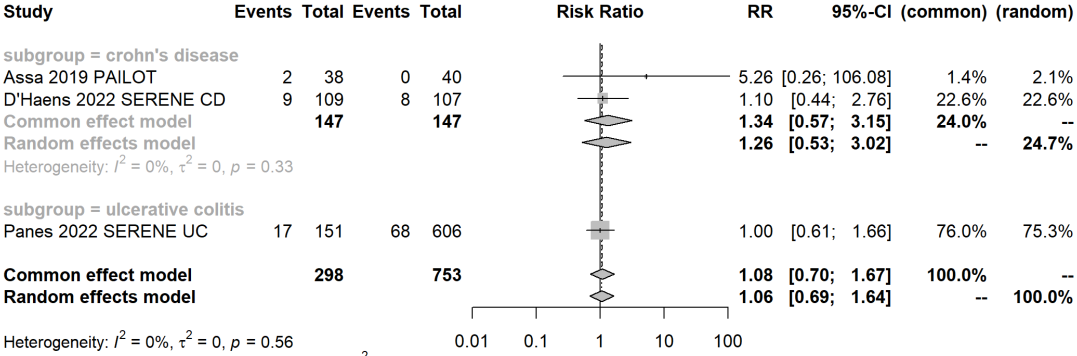

Heterogeneity: $I^2 = 0\%$, $\tau^2 = 0$, $p = 0.56$
Test for subgroup differences (common effect): $\chi^2_1 = 0.33$, df = 1 ($p = 0.57$)
Test for subgroup differences (random effects): $\chi^2_1 = 0.20$, df = 1 ($p = 0.66$)

Supplement 7: Forest plots for analyses of therapeutic drug monitoring of infliximab for maintenance

Sustained disease control or sustained remission

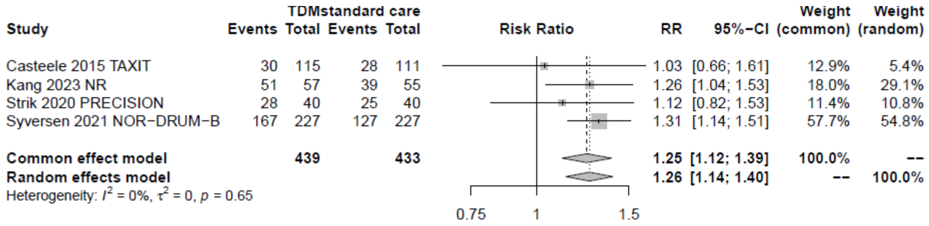

Sustained disease control

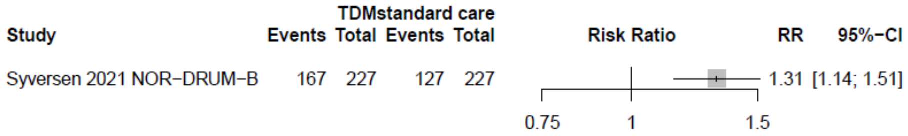

Sustained remission

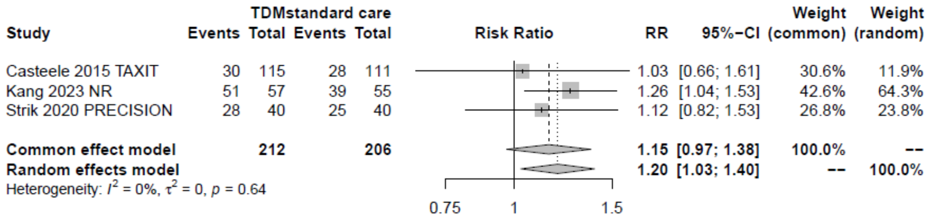

Remission

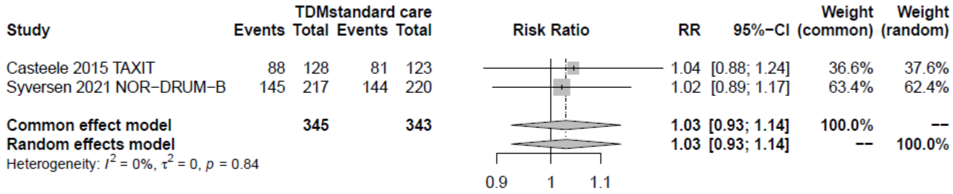

Disease worsening

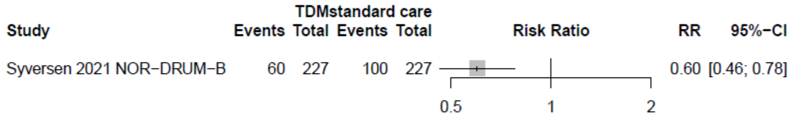

Serious adverse events

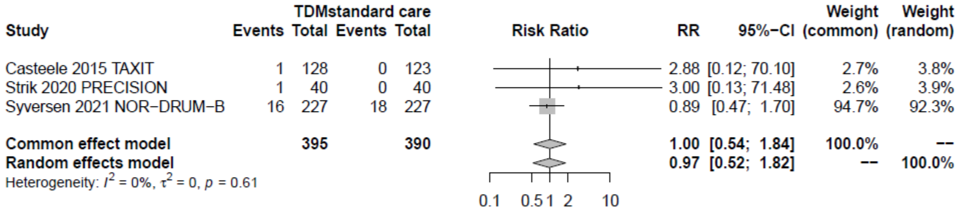

Adverse events leading to discontinuation of therapy

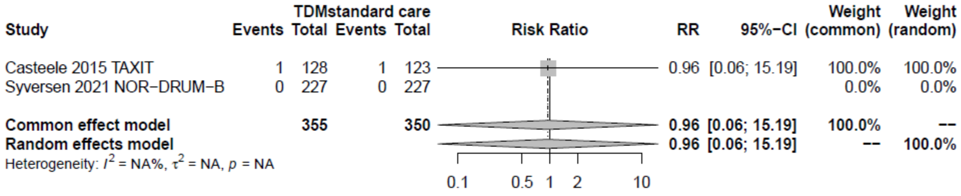

Anti-drug antibodies

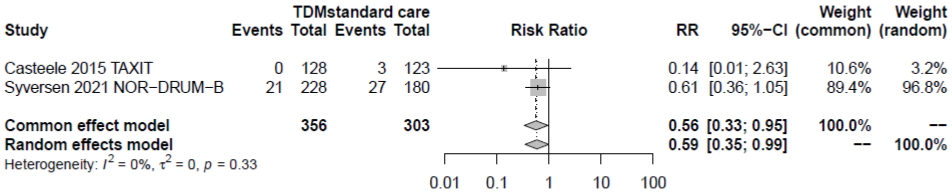

Supplement 8: Subgroup analyses based on age

Patient group	Drug	Outcome	Subgroup	Studies	Participants	RR (95% CI)	I2	P value for interaction
**Maintenance**	Infliximab	Sustained remission or disease control	children	1	112	1.26 (1.04, 1.53)	NA	0.94
			adult	3	760	1.25 (1.09, 1.43)	0	
		Sustained remission	children	1	112	1.26 (1.04, 1.53)	NA	0.37
			adult	2	306	1.09 (0.84, 1.41)	0	
	Adalimumab	Remission	children	1	78	1.45 (1.09, 1.92)	NA	0.02
			adult	2	555	0.97 (0.82, 1.15)	0	
		Serious adverse events	children	1	78	1.05 (0.28, 3.91)	NA	0.61
			adult	2	975	0.73 (0.46, 1.17)	0	
		Adverse events leading to discontinuation of therapy	children	1	78	5.26 (0.26, 106.08)	NA	0.29
			adult	2	975	1.03 (0.66, 1.6)	0	

Supplement 9: Forest plots for analyses of therapeutic drug monitoring of adalimumab for maintenance

Sustained disease control or sustained remission

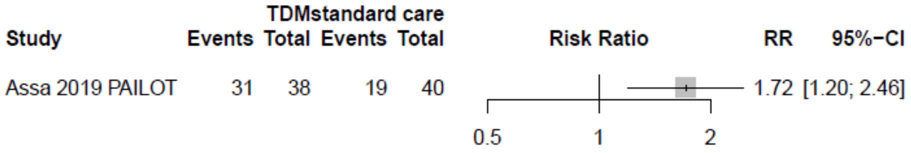

Sustained remission

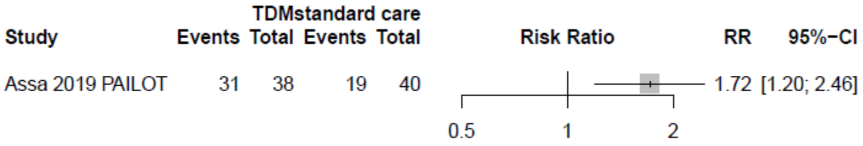

Remission

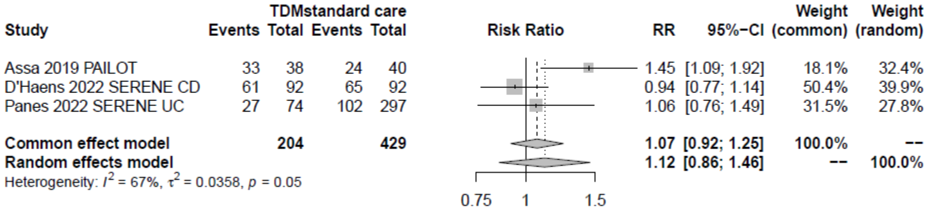

Disease worsening

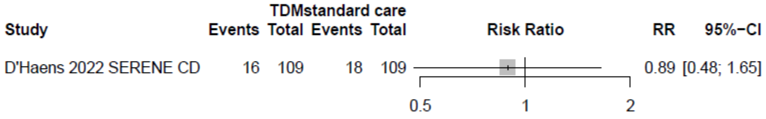

Serious adverse events

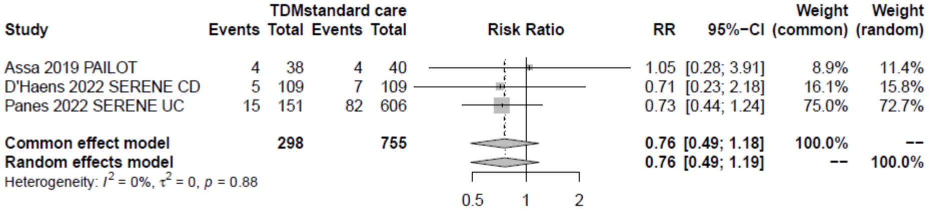

Adverse events leading to discontinuation of medical therapy

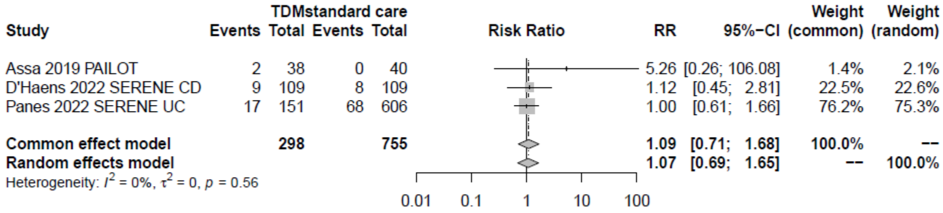

Anti-drug antibodies

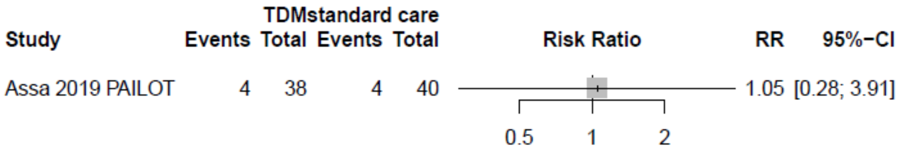

Supplement 10: Evaluation of the credibility of the subgroup effect based on age using the ICEMAN tool

**1: Is the analysis of effect modification based on comparison within rather than between trials?**	**Completely between**	Mostly between or unclear	Mostly within	Completely within
**2: For within-trial comparisons, is the effect modification similar from trial to trial?**	Definitely not similar	Probably not similar or unclear	Mostly similar	Definitely similar
**3: For between-trial comparisons, is the number of trials large?**	**Very small**	Rather small or unclear	Rather large	Large
**4: Was the direction of effect modification correctly hypothesized a priori?**	Definitely no	**Probably no or unclear**	Probably yes	Definitely yes
**5: Does a test for interaction suggest that chance is an unlikely explanation of the apparent effect modification?**	Chance a very likely explanation	**Chance a likely explanation or unclear**	Chance may not explain	Chance an unlikely explanation
**6: Did the authors test only a small number of effect modifiers or consider the number in their statistical analysis?**	Definitely no	Probably no or unclear	Probably yes	**Definitely yes**
**7: Did the authors use a random effects model?**	Definitely no	Probably no or unclear	Probably yes	**Definitely yes**
**8: If the effect modifier is a continuous variable, were arbitrary cut points avoided?**	Definitely no	Probably no or unclear	Probably yes	Definitely yes
