# Supplementary material for: Proactive therapeutic drug monitoring of biologic drugs in patients with inflammatory bowel disease, inflammatory arthritis, and psoriasis: systematic review and meta-analysis
Source: BMJ Med. 2024 Oct 28;3(1):e000998. doi: 10.1136/bmjmed-2024-000998 (PMC11579540; doi:10.1136/bmjmed-2024-000998)
Supplement: Supplementary file 2 [file bmjmed-2024-000998.reviewer_comments.pdf]

PEER REVIEW HISTORY

BMJ Medicine publishes all reviews undertaken for accepted manuscripts. Reviewers are asked to complete a checklist review form and are provided with free text boxes to elaborate on their assessment. These free text comments are reproduced below.

This paper was submitted to a another journal from BMJ but declined for publication following peer review. The authors addressed the reviewers' comments and submitted the revised paper to BMJ Medicine. The paper was subsequently accepted for publication at BMJ Medicine.

ARTICLE DETAILS

**TITLE (PROVISIONAL)**	Proactive therapeutic drug monitoring of biologic drugs in patients with inflammatory bowel disease, inflammatory arthritis, and psoriasis: A systematic review and meta-analysis
**AUTHORS**	Zeraatkar, Dena; Pitre, Tyler; Kirsh, Sarah; Jassal, Tanvir; Ling, Michael; Hussain, Muizz; Couban, Rachel; Kawano-Dourado, Leticia; Kristianslund, Eirik; Olav Vandvik, Per

VERSION 1 - REVIEW

**REVIEWER NAME**	Adam Cheifetz
**REVIEWER AFFILIATION**	BIDMC
**REVIEWER CONFLICT OF INTEREST**	None
**DATE REVIEW RETURNED**	05-May-2024

**GENERAL COMMENTS**	Overall very well done systematic review and meta-analysis. I just don't think it adds enough to the literature for BMJ, but will leave that up to editors.<br><br>Some comments:<br>Major points:<br>1. The TAILORIX study doesn't refer to induction. In this study infliximab optimization was only allowed from week 14 and onward. So, there is no need for a meta-analysis regarding the role of proactive TDM during induction therapy if only one study (NOR-DRUM A) is only available. Consequently, the relevant section should be deleted.<br>2. The title includes 'adults', although the PAILOT study included in the meta-analysis refers to a pediatric population. So, the word 'adults' should be deleted from the title.<br>3. Page 13, lines 447, 448, the text has to be revised as ref. 33 actually showed that 'compared to SOC, proactive TDM was associated with significant benefit in reducing treatment failure. Compared to reactive TDM, proactive TDM led to a significant reduction in hospitalisation and treatment failure.'<br><br>Minor points:<br>1. Page 13, line 445 please add another two ongoing TDM RCTs (NCT04835506 and ACTRN12621000023853)<br>2. Page 14, lines 479, 480, proactive TDM has been proven cost effective (DOI: 10.3390/pharmaceutics14051009)

**REVIEWER NAME**	Xiaoling Cai
**REVIEWER AFFILIATION**	Peking University People's Hospital
**REVIEWER CONFLICT OF INTEREST**	None
**DATE REVIEW RETURNED**	05-May-2024

**GENERAL COMMENTS**	Dena Zeraatkar et al. present here an interesting systematic review and meta-analysis of all randomized trials comparing proactive therapeutic drug monitoring with standard care in adult patients with inflammatory bowel disease, inflammatory arthritis, and psoriasis. The paper was well written and accessible. However, the conclusions were low certainty evidence. I have several comments regarding the manuscript:<br><br>Please provide the research question using PICO formulation. Pleased be more explicit about the population included.<br><br>This study included randomized trials from inception to December 8, 2022. I encourage authors to updated the literature search.<br><br>I don't think it's appropriate that this current research grouped inflammatory bowel disease, inflammatory arthritis, and psoriasis together. Previous study found when restricted to inflammatory bowel disease, no evidence of benefit with proactive therapeutic drug monitoring was found.<br><br>I would suggest to strengthen the point of limitations. The duration of follow-up across trials was too short to assess the long-term effects of proactive therapeutic drug monitoring. Did authors do subgroup analyses based on follow-up time?

VERSION 1 – AUTHOR RESPONSE

Reviewer 1

Comments:
Overall very well done systematic review and meta-analysis. I just don't think it adds enough to the literature for BMJ, but will leave that up to editors.

Some comments:
Major points:
1. The TAILORIX study doesn't refer to induction. In this study infliximab optimization was only allowed from week 14 and onward. So, there is no need for a meta-analysis regarding the role of proactive TDM during induction therapy if only one study (NOR-DRUM A) is only available. Consequently, the relevant section should be deleted.

Our response: Our categorization of trials reflects the definition used by the parallel guideline panel regarding induction and maintenance. We considered induction to refer to the period of treatment from active disease to(wards) remission. The TAILORIX trial includes patients “with active luminal Crohn’s Disease”. Patients were randomized at 3 months. Based on available evidence that suggests that the median time to remission for patients with active disease exceeds 3 months, we anticipate that most patients included in this trial would be in a state of active disease.

We have revised to clarify.

Revision	Page	Line
**Induction of therapy:** The initiation of biologic drugs when patients are in a state of active disease, with the aim of treatment to achieve disease control, preferably by reaching a state of remission.<br><br>**Maintenance of therapy:** The use of biologic drugs when patients are in a state of disease control to avoid disease worsening.	5	Box 1: 131
Another trial of adults with active Crohn's disease randomized patients at 14 weeks following the first dose of infliximab to proactive therapeutic drug monitoring or standard care (66). We grouped this trial with those reporting on induction as it initially recruited patients with active disease and we deemed 14 weeks insufficient for most patients to achieve remission prior to randomization.	10	326 to 329

2. The title includes 'adults', although the PAILOT study included in the meta-analysis refers to a pediatric population. So, the word 'adults' should be deleted from the title.

Our response: We have revised as suggested.

Revision	Page	Line
Proactive therapeutic drug monitoring of biologic drugs in patients with inflammatory bowel disease, inflammatory arthritis, and psoriasis: A systematic review and meta-analysis	1	1 to 3

3. Page 13, lines 447, 448, the text has to be revised as ref. 33 actually showed that 'compared to SOC, proactive TDM was associated with significant benefit in reducing treatment failure. Compared to reactive TDM, proactive TDM led to a significant reduction in hospitalisation and treatment failure.'

Our response: We have revised as suggested.

Revision	Page	Line
Previous systematic reviews have reported inconsistent results addressing the effects of proactive therapeutic drug monitoring (31, 33). These reviews, however, were restricted to inflammatory bowel disease and pooled results of trials together across biologic drugs and induction and maintenance.	14	477 to 479

Minor points:
1. Page 13, line 445 please add another two ongoing TDM RCTs (NCT04835506 and ACTRN12621000023853)

Our response: We have revised as suggested.

Revision	Page	Line
We did not find any trials addressing therapeutic drug monitoring of biologic drugs other than infliximab adalimumab, and etanercept, though there are ongoing trials of therapeutic drug monitoring for other biologic drugs (NCT03895879). There are also additional ongoing trials of therapeutic drug monitoring of infliximab and	14	469 to 475

adalimumab, which may improve the precision of estimates and may be able to clarify as to whether the effects of therapeutic drug monitoring are consistent across immune-mediated inflammatory diseases (NCT04775732, NCT02508012, NCT03261102, NCT04835506, ACTRN12621000023853).		

2. Page 14, lines 479, 480, proactive TDM has been proven cost effective (DOI: 10.3390/pharmaceutics14051009)

Our response: We have revised as suggested.

**Revision**	**Page**	**Line**
Practitioners and other decision-makers, however, should be mindful about the cost-effectiveness of therapeutic drug monitoring and other additional challenges related to its implementation. While proactive therapeutic drug monitoring has been reported largely cost-effective, cost-effectiveness analyses have primarily focused on inflammatory bowel disease and North America and Western Europe (77). The cost-effectiveness of therapeutic drug monitoring in other settings and for other diseases is unclear.	15	508 to 513

Reviewer 2

Dena Zeraatkar et al. present here an interesting systematic review and meta-analysis of all randomized trials comparing proactive therapeutic drug monitoring with standard care in adult patients with inflammatory bowel disease, inflammatory arthritis, and psoriasis. The paper was well written and accessible. However, the conclusions were low certainty evidence. I have several comments regarding the manuscript:

Please provide the research question using PICO formulation. Pleased be more explicit about the population included.

Our response: We have revised as suggested.

**Revision**	**Page**	**Line**
We present a systematic review and meta-analysis of randomized trials addressing the comparative efficacy and safety of proactive therapeutic drug monitoring of biologics during induction or maintenance in patients with immune-mediated inflammatory diseases, including inflammatory bowel disease, inflammatory arthritis, and psoriasis.	4	120 to 123

This study included randomized trials from inception to December 8, 2022. I encourage authors to updated the literature search.

Our response: Please see our response to the comment above encouraging an updated search.

I don't think it's appropriate that this current research grouped inflammatory bowel disease, inflammatory arthritis, and psoriasis together. Previous study found when restricted to inflammatory bowel disease, no evidence of benefit with proactive therapeutic drug monitoring was found.

Our response: Please see our response to a comment above addressing pooling of results across diseases. Further, one review restricted to inflammatory bowel disease did indeed find evidence of benefit with therapeutic drug monitoring.

> Sethi S, Dias S, Kumar A, Blackwell J, Brookes MJ, Segal JP. Meta-analysis: The efficacy of therapeutic drug monitoring of anti-TNF-therapy in inflammatory bowel disease. Aliment Pharmacol Ther. 2023 Jun;57(12):1362-1374. doi: 10.1111/apt.17313. Epub 2022 Dec 9. PMID: 36495020.

I would suggest to strengthen the point of limitations. The duration of follow-up across trials was too short to assess the long-term effects of proactive therapeutic drug monitoring. Did authors do subgroup analyses based on follow-up time?

Our response: The sparsity of data precluded subgroup analyses based on follow-up time. We have revised to further emphasize the limited duration of follow-up across trials.

Revision	Page	Line
The duration of follow-up across trials was limited to one year, making the long-term effects of proactive therapeutic drug monitoring uncertain. This is a major shortcoming, as these diseases are chronic, requiring patients to use biologics over extended periods, with flares and disease worsening often occurring after prolonged use of biologics (73).	13	432 to 435

VERSION 2 – REVIEW

**REVIEWER NAME**	Chanchlani, Neil
**REVIEWER AFFILIATION**	Bristol Royal Hospital for Children
**REVIEWER CONFLICT OF INTEREST**	None
**DATE REVIEW RETURNED**	20-Jul-2024

**GENERAL COMMENTS**	Overall<br>I think this is OK now. They have updated and expanded the search, and provided decent and thoughtful replies to concerns. Conclusions have been substantially tempered and limitations beefed up. I think this is less prone to criticism from specialists now. Do I really think it adds much more than the two IBD SR/MAs - probably not very much - but it is such a hot topic that it will be cited, and at least comes a couple years later than the other 2. Also, I think the Rapid Recc is going ahead, so another reason to perservere with this linked piece. Though I haven't been asked to review the revision of the Rapid Recc yet (Rafael has), Tom was keen to get something out of it.<br><br>A question for you:<br>Re the million dollar/pound question - 'should these disesases have been all combined?' I understand their theory and rationale for their approach. I guess its all hinges on Supplement 6, which does not show a significant p value for interaction (is that what the basis of 'no difference between disease subtypes' is dependent on?) Forgive my statistical ignorance, why would they not do a Forest plot like they did for the other supplementary so we can visualise this?

	Two minor quips, that can be ignored or requested post acceptance<br>Box 1<br>Proactive definition - For clarity, in box 1, I would suggest inclusion of 'irrespective of symptoms/disease worsening'<br><br>Induction/maintenance of therapy - traditionally, these phases of care have timelines attributed to them (eg - first 12 - 20 weeks of starting [or being re-induced] with a biologic, >20 weeks of starting a biologic for maintenance). Slightly odd to just refer to state of disease without a timeline - I think they need to justify LACK of inclusion of timeline more than they have in Box 1.<br><br>This is important as per rev 1, who picked up on this with respect to the TAILORIX trial not truly being reflective of disease induction. Au have responded that patients were still in active disease (because patients are > 3 months) so they considered it 'induction', rather than a timeline cut-off. Kinda weird and unconventional, but OK that is their prerogative. They have written it clearly in the manuscript, which I think will help avoid pushback/rapid response.<br><br>Re whether there is a clear message for generalists<br>I'm not sure this was ever going to have a clear, actionable take-home to non-specialists, who do not often check these levels/antibodies for patients. In the clinical context where GPs perform bloods on specialists' behalf (increasingly important with the increasing use of subcutanous infliximab, thereby avoiding regular/opportunistic secondary care reviews altogether), they should feel empowered to tick 'infliximab drug/antibody' as well as the regular bloods (inflammation/renal/liver, FBC). But they do not act on the results without specialist input.<br><br>For derm/rheum/gastro specialists, the headline is to perform therapeutic drug monitoring opportunistically at regular infusions (infliximab), and not wait for patients to deteriorate before checking levels/antibodies. This means you are one step ahead, not behind, when making dose adjustments to avoid loss of response to these drugs.<br><br>I think that message is relatively preserved. But I also think reinforcing this clinical message(s) can also be be the priority of the Rapid Recc,

**REVIEWER NAME**	Perera, Rafael
**REVIEWER AFFILIATION**	University of Oxford, Primary Care Health Sciences
**REVIEWER CONFLICT OF INTEREST**	None
**DATE REVIEW RETURNED**	25-Jul-2024

**GENERAL COMMENTS**	Stats Review<br><br>The current manuscript presents results from a systematic review of all randomised controlled trials evaluating the impact of proactive therapeutic drug monitoring for three biologic drugs (infliximab, adalimumab, etanercept; as the authors have not found evidence for other biologic drugs). Their main finding is that the evidence available provides low or very low certainty of a) small or no effect for those outcomes evaluated, except for b) a potential increase in the proportion of patients with sustained disease control or remission

	in those using proactive therapeutic monitoring for maintenance with infliximab.<br><br>There are some minor clarifications and adjustments that would be relevant before a recommendation for publication can be given.<br><br>• Preprints were also accepted (as well as those with full publication in peer-reviewed journals) in the eligibility criteria. Was there an exploration (sensitivity analysis) of the potential differences observed between the evidence from preprints and that from fully published studies?<br>• Were the disease-specific activity scores considered part of the inclusion/exclusion criteria? Are these scores commonly recognised as the activation thresholds? Were there trials not included as they did not fit with these thresholds?<br>• Generally, for Random Effects models, the HKJS method is recognised as providing better 95%CI coverage than REML. Please justify your choice or provide HKSJ estimates.<br>• Although i-squared is more commonly used to report general levels of heterogeneity, the actual estimate of heterogeneity is tau-squared, which you calculate and many methodologists prefer. The tau-squared is an actual estimation of heterogeneity (and not the i-squared). It would be useful to adjust this in your methods section regarding reporting heterogeneity across studies.<br>• You use meta-regression to evaluate differences within subgroups. The result for this is presented as a p-value (test of interaction). Please clarify how you carried out/parametrised this meta-regression. Were the categories treated as different factors or using some form of numerical ordering? Please expand on the methods used, as this is currently unclear.<br>• In the Results section (lines 406- 408), there is a copy/paste mistake here with a discussion of results for infliximab when this section is about adalimumab.

VERSION 2 – AUTHOR RESPONSE

Reviewer: 1
Prof. Rafael Perera, University of Oxford
Comments to the Author
bmjmed-2024-000998 - Proactive therapeutic drug monitoring of biologic drugs in patients with inflammatory bowel disease, inflammatory arthritis, and psoriasis: A systematic review and meta-analysis

Stats Review

The current manuscript presents results from a systematic review of all randomised controlled trials evaluating the impact of proactive therapeutic drug monitoring for three biologic drugs (infliximab, adalimumab, etanercept; as the authors have not found evidence for other biologic drugs). Their main finding is that the evidence available provides low or very low certainty of a) small or no effect for those outcomes evaluated, except for b) a potential increase in the proportion of patients with sustained disease control or remission in those using proactive therapeutic monitoring for maintenance with infliximab.

There are some minor clarifications and adjustments that would be relevant before a recommendation for publication can be given.

• Preprints were also accepted (as well as those with full publication in peer-reviewed journals) in the eligibility criteria. Was there an exploration (sensitivity analysis) of the potential differences observed between the evidence from preprints and that from fully published studies?

Our response: We did not identify any eligible preprints. We have revised to clarify.

**Revision**	**Page**	**Line**
All trials were published in peer reviewed journals. Results from one trial were only available as a conference abstract (71).	10	313 to 314

• Were the disease-specific activity scores considered part of the inclusion/exclusion criteria? Are these scores commonly recognised as the activation thresholds? Were there trials not included as they did not fit with these thresholds?

Our response: We did not consider the disease-specific activity scores as part of our eligibility criteria. These scores, and the corresponding thresholds, are commonly recognized in the field. Our authorship group, including clinical experts in this area, are unaware of additional or alternative scoring systems or thresholds.

We did not come across any trials that used alternative scoring systems or thresholds to define remission, sustained remission, disease control, or disease worsening.

We have revised to clarify.

**Revision**	**Page**	**Line**
These scoring systems and thresholds are widely recognized and represent systems and thresholds used to operationalize disease activity both in clinical practice and in research (23, 24, 37, 47-50).	6	195 to 196

• Generally, for Random Effects models, the HKJS method is recognised as providing better 95%CI coverage than REML. Please justify your choice or provide HKSJ estimates.

Our response: We acknowledge that the HKSJ method has shown superior performance to conventional methods for calculating confidence intervals. Despite this, HKSJ has had slower than anticipated adoption in systematic reviews.

We used the standard method for the calculation of confidence intervals and used the REML method to estimate heterogeneity, which can also be combined with the HKSJ method. Our rationale for this approach was that most systematic reviews use conventional methods to calculate confidence intervals and simulation studies suggest the REML heterogeneity estimator is among the best heterogeneity estimators, outperforming other methods like the Dersimonian-Laird method.

> Veroniki AA, Jackson D, Bender R, Kuss O, Langan D, Higgins JPT, Knapp G, Salanti G. Methods to calculate uncertainty in the estimated overall effect size from a random-effects meta-analysis. Res Synth Methods. 2019 Mar;10(1):23-43. doi: 10.1002/jrsm.1319. Epub 2018 Oct 9. PMID: 30129707.

We are fairly confident that using the HKSJ method will not importantly affect our estimates, since the biggest difference between results from the HKSJ method and the conventional method for calculating confidence intervals occurs in meta-analyses with high heterogeneity, which was not observed in the present systematic review.

Further, the HKSJ method can also produce counterintuitive results, such as when heterogeneity is very low or in meta-analyses of rare events.

Röver C, Knapp G, Friede T. Hartung-Knapp-Sidik-Jonkman approach and its modification for random-effects meta-analysis with few studies. BMC Med Res Methodol. 2015 Nov 14;15:99. doi: 10.1186/s12874-015-0091-1. PMID: 26573817; PMCID: PMC4647507.

If the editors and reviewers feel strongly, we can revise to use the HKSJ method.

• Although i-squared is more commonly used to report general levels of heterogeneity, the actual estimate of heterogeneity is tau-squared, which you calculate and many methodologists prefer. The tau-squared is an actual estimation of heterogeneity (and not the i-squared). It would be useful to adjust this in your methods section regarding reporting heterogeneity across studies.

Our response: We agree with the reviewer's suggestion. We report tau^2 for all meta-analyses. While tau^2 is not reported in the main manuscript, we report tau^2 for all analyses in forest plots, which are available in the manuscript supplement. We prioritized reporting I^2 values over tau^2 because I^2 is independent of the metric of the outcome and most familiar to evidence users.

• You use meta-regression to evaluate differences within subgroups. The result for this is presented as a p-value (test of interaction). Please clarify how you carried out/parametrised this meta-regression. Were the categories treated as different factors or using some form of numerical ordering? Please expand on the methods used, as this is currently unclear.

Our response: We have revised to clarify. We performed subgroup analyses to investigate differences in the effects of proactive therapeutic drug monitoring based on risk of bias, disease, and age (trials of adults vs. children). We did not perform meta-regressions. Since we did not perform meta-regressions, we did not use any numerical ordering.

Revision	Page	Line
To test for subgroup effects based on risk of bias, type of disease, concomitant immunosuppression, and exposure to previous biologic drugs, we performed subgroup analyses and evaluated the credibility of subgroup effects using the ICEMAN tool (64).	9	275 to 277

• In the Results section (lines 406- 408), there is a copy/paste mistake here with a discussion of results for infliximab when this section is about adalimumab.

Our response: We have revised as suggested.

Revision	Page	Line
We anticipated that the effect of therapeutic drug monitoring of adalimumab may be different in trials at low versus high risk of bias and based on disease and age. We did not find evidence that the effects of therapeutic drug monitoring of adalimumab were different in trials at low versus high risk of bias or based on disease, though effects across disease subgroups were imprecise (Supplements 5 and 6).	12	412 to 415

If you have any competing interests: None

Editor(s)' Comments to Author:

Clinical Editor (John Fletcher):
Neil Chanchlani, BMJ clinical editor, made the following requests for revision:
* Box 1 Proactive definition - For clarity, in box 1, I would suggest inclusion of 'irrespective of symptoms/disease worsening'

Our response: We have revised as suggested.

Revision	Page	Line
**Proactive therapeutic drug monitoring:** The scheduled measurement of serum drug concentrations and anti-drug antibodies to optimize individual patient dosage regimens and achieve target drug serum levels irrespective of symptoms or disease worsening (20, 22).	5	136

* For Box 1 please define the time windows for induction and maintenance or alternatively say why you have not included a time (eg - first 12 - 20 weeks of starting [or being re-induced] with a biologic, >20 weeks of starting a biologic for maintenance).

Our response: We avoid specifying a time frame because the time frame varies across diseases. Instead, we differentiate between induction and maintenance based on the objective of treatment, with induction being to induce disease remission and maintenance to maintain disease remission. We have revised to clarify.

Revision	Page	Line
**Induction of therapy**: The initiation and use of biologic drugs when patients are in a state of active disease, with the aim of treatment to achieve disease control, preferably by reaching a state of remission. The duration of induction therapy may vary across diseases. We differentiate induction from maintenance based on the treatment objective: induction aims to achieve remission or disease control, while maintenance focuses on sustaining that state.	5	136

VERSION 3 – REVIEW

**REVIEWER NAME**	Perera, Rafael
**REVIEWER AFFILIATION**	University of Oxford, Primary Care Health Sciences
**REVIEWER CONFLICT OF INTEREST**	None
**DATE REVIEW RETURNED**	16-Sep-2024

**GENERAL COMMENTS**	I want to thank the authors for replying to my comments. These have now been adequately addressed, and I believe the current version meets the right standard for publication in BMJ Medicine.
